# Defect-complementation homologous recombination: A novel strategy for precise genome engineering of virulent phages

**DOI:** 10.1016/j.synbio.2025.11.002

**Published:** 2025-11-17

**Authors:** Hailin Zhang, Yueyue Song, Wenyue Liu, Xiaoqing Zheng, Xiaodong An, Chao Li, Weihua Chen, Hailong Wang, Yuran Zhang

**Affiliations:** a School of Life Sciences, Jining Medical University, No. 669 Xueyuan Road, Donggang District, Rizhao, Shandong Province, 276826, China; b Rizhao Center for Disease Control and Prevention, No. 136, Beijing Road, Donggang District, Rizhao, Shandong Province, 276826, China; c Key Laboratory of Molecular Biophysics of the Ministry of Education, Hubei Key Laboratory of Bioinformatics and Molecular Imaging, Center for Artificial Intelligence Biology, Department of Bioinformatics and Systems Biology, College of Life Science and Technology, Huazhong University of Science and Technology, Wuhan, Hubei, China; d State Key Laboratory of Microbial Technology, Institute of Microbial Technology, Helmholtz International Lab for Anti-infectives, Shandong University–Helmholtz Institute of Biotechnology, Shandong University, Qingdao, Shandong, 266237, China; e Rizhao Research Institute of Shandong University, Rizhao, Shandong, 276800, China

**Keywords:** Genetic complementation, Phage genome engineering, Cre-lox, Phage genetic manipulation, Homologous recombination

## Abstract

Engineered bacteriophages (phages) have been developed to overcome the limitations of natural phage therapies and serve as precision-targeted agents against drug-resistant bacterial infections. However, their application has been constrained by the low efficiency of existing genome-editing tools, largely because of the absence of effective selection markers. This study proposed a novel strategy, termed defect-complementation homologous recombination (DCHR), for precise phage genome editing. In this approach, CRISPR-Cas9 cleaves a donor plasmid in host cells to release a linear donor template carrying homology arms, an essential phage gene used as a selection marker, and two *lox* sites. The donor template undergoes homologous recombination with the genome of essential gene-deficient phage, thereby enabling targeted genome modifications. Using DCHR, we successfully generated large genomic deletions (1.48-kb *gp0.4–0.7* and 1.02-kb *gp4.3–4.7*), achieved gene insertion (3.08-kb *lacZ*), and introduced a single-base substitution (TGA to TAA) in the stop codon of *gp9* within the same T7 phage genome, all with 100 % accuracy. The significant advantages of DCHR are as follows: (i) High-efficiency screening: Only progeny phages derived from successful homologous recombination retain viability and replicative capacity, thereby greatly simplifying recombinant isolation. (ii) Editing flexibility: Unlike CRISPR-Cas systems, DCHR cannot be constrained by protospacer adjacent motif dependence and allows modifications across diverse genomic loci. (iii) High recombination efficiency: DCHR can achieve a recombinant phage titer of 3.1 × 10^5^ PFU mL^−1^ (plaque-forming units per mL) without relying on exogenous homologous recombination systems. In summary, DCHR demonstrates potential as a precise and efficient general genome-editing tool that facilitates design of engineered phages and advances functional genomic studies.

## 1 Introduction

The global overuse of antimicrobial agents combined with inadequate regulatory oversight has caused a growing public health crisis of antimicrobial resistance [1]. As viruses that specifically infect bacteria, virulent bacteriophages (phages) eradicate drug-resistant pathogens through their strictly lytic lifecycle [2].Thus, phage therapy has emerged as a promising strategy against multidrug-resistant infections and has attracted increasing attention in recent years [[3], [4], [5]]. However, the clinical application of natural phages faces substantial challenges, including limited bactericidal efficacy, a narrow host range, and the potential presence of virulence-associated genes in their genomes, which can hinder therapeutic translation [6]. Engineered phages developed through precision genome editing provide a strategy to overcome the biological limitations of natural phages, thereby advancing phage therapy [7,8]. However, the successful artificial modification of phages requires a comprehensive understanding of the genomic structure and function, followed by the utilization of precise engineering strategies and rational *de novo* designs. The attainment of these objectives relies heavily on genome-editing technologies that are precise, efficient, and practical.

Several strategies for phage genome editing have been reported [9], including yeast-based assembly [10,11], *de novo* synthesis technology [[12], [13], [14]], SMART (Splitting, Modifying, Assembling, and Rebooting) [15], homologous recombination [16,17], BRED (Bacteriophage Recombineering of Electroporated DNA) [18], and CRISPR (Clustered Regularly Interspaced Short Palindromic Repeats)-Cas (CRISPR-associated proteins) systems [19,20]. Despite their diversity, these approaches share certain limitations in analyzing phage genomic architecture and functional annotation. Yeast-based assembly and *de novo* synthesis technologies generate engineered phages by synthesizing phage genomic fragments using PCR or chemical synthesis, followed by fragment assembly into functional genomes. While these methods can achieve single-nucleotide precision in genome editing, inevitable random mutations during synthesis can disrupt genotype-phenotype correlations. The SMART method facilitates high-fidelity editing via segmental cloning of phage genomes and *in situ* plasmid modification, while the inherent toxicity of phage genomes to host cells poses major challenges for segmental cloning. Homologous recombination and BRED both rely on allelic recombination to enable precise gene editing. However, given the low recombination frequency (10^−4^ to 10^−8^) of homology-directed repair [16,17], effective selection markers are indispensable for isolating recombinant phages. This requirement is particularly problematic for virulent phages, which rapidly lyse host cells, rendering conventional antibiotic resistance-based positive selection systems ineffective. This contributes to laborious isolation procedures. Although CRISPR-Cas systems can be employed as counter-selection tools to enhance recombination accuracy (∼20 %) [21,22], their efficiency and precision are compromised by inherent limitations, such as low nuclease activity in certain microbial hosts [23], potential off-target effects, and strict dependence on protospacer adjacent motifs (PAMs) [24,25]. To date, no available tool integrates high fidelity, efficiency, and operational simplicity to fully satisfy the demands of engineered phage design and development.

In this study, we developed a novel genome-editing method, termed defect-complementation homologous recombination (DCHR), to address the lack of selection markers in conventional homologous recombination for virulent phage genome engineering. This strategy is based on homologous recombination and employs the complementation of essential phage genes to achieve precise genome modification. Specifically, CRISPR-Cas9-mediated cleavage of a donor plasmid in host cells generates linear donor templates that contain homology arms (HAs), an essential phage gene serving as a selection marker, and *lox66/lox71* recombination sites. These donor templates subsequently mediate targeted genomic modifications through homologous recombination with phage genomic DNA lacking the essential gene. Subsequently, Cre-lox site-specific recombination between the *lox66* and *lox71* sites mediates excision of the selection marker, enabling iterative rounds of editing. Finally, after the editing process, the essential gene is restored to its native genomic locus via homologous recombination, thereby recovering its wild-type genotype. Using the DCHR method, gene deletion, insertion, and point mutation were performed within the same T7 phage genome. DCHR demonstrated high recombination efficiency independent of exogenous homologous recombination systems and electroporation for genomic DNA delivery, underscoring its potential as a precise and general genome-editing tool.

## 2 Materials and methods

### 2.1 Bacteria culture conditions

*Escherichia coli* strains (Table S1) were cultured at 30 °C or 37 °C in LB medium supplemented with the appropriate antibiotics: chloramphenicol (*Cm*, 15 μg mL^−1^), ampicillin (Amp, 100 μg mL^−1^, kanamycin (Kan, 5 μg mL^−1^), or tetracycline (Tet, 5 μg mL^−1^).

### 2.2 The workflow diagram of DCHR

*Enterobacteria* phage T7 is a virulent phage that specifically infects *E. coli* and serves as a well-established model system in molecular biology [26]. Previous studies have identified its nonessential gene regions (Table S2) [15,27]. In this study, T7 was used as the experimental model, and the 1.04-kb essential gene *gp10* (encoding the T7 major capsid protein) was selected as the marker gene. The *gp10*-deficient T7 phage was then adopted as the initial strain to demonstrate the DCHR workflow.

**Fig. 1:**
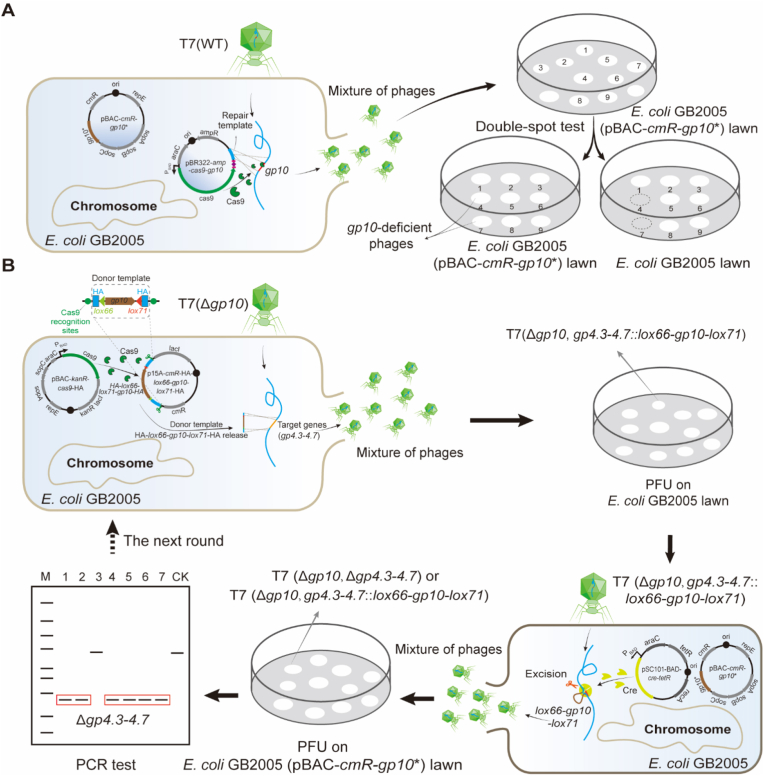
Workflow of the DCHR strategy. (A) Construction of the *gp10*-deficient T7 phage. T7(WT) represents the wild-type T7 phage. (B) Schematic overview of DCHR workflow. The HA-*lox66-gp10-lox71*-HA donor template, released from CRISPR-Cas9-guided cleavage of the p15A donor plasmid, underwent homologous recombination with the T7 (Δ*gp10*) genomic DNA in *E. coli* GB2005, replacing the targeted genes (*gp4.3*–*4.7*). The recombinant T7 phages (Δ*gp10*, *gp4.3*–*4.7::lox66-gp10-lox71*) were isolated on *E. coli* GB2005 lawns and subsequently used to infect *E. coli* GB2005 (harboring pSC101-BAD-*cre-tetR* and pBAC-*cmR-gp10*∗ plasmids) that pre-expressed Cre recombinase and functional Gp10 protein. Following Cre-lox-mediated site-specific recombination, *gp10* was excised, and recombinant T7 phages (Δ*gp10*, Δ*gp4.3*–*4.7*) were isolated on *E. coli* GB2005 (pBAC-*cmR-gp10*∗) lawns and verified by PCR and Sanger sequencing. The phages could then proceed to the next round of DCHR. White dots indicate plaques, whereas dashed lines represent no detectable plaques.

Prior to implementing DCHR, the construction of a *gp10*-deficient T7 phage was required. CRISPR-Cas9-assisted engineering was employed to generate this mutant [28] (Fig. 1A). Two plasmids were constructed and introduced into *E. coli* GB2005 cells. The first was a pBR322-based plasmid (designated pBR322-*ampR-cas9-gp10*) containing three functional components: (i) an arabinose-inducible expression system for Cas9 nuclease expression, (ii) a DNA sequence encoding a single guide RNA (sgRNA) targeting the wild-type *gp10* gene, and (iii) a 1.0-kb repair template consisting of two 500-bp HAs flanking *gp10* to mediate homology-directed repair. The second plasmid was an engineered bacterial artificial chromosome (BAC, designated pBAC-*cmR-gp10*∗) expressing the Gp10 protein. To ensure functional specificity, all codons of *gp10* in the BAC plasmid required synonymous mutations to achieve two critical goals: avoiding recognition by the inducible sgRNA-guided Cas9 nuclease and preventing wild-type *gp10* from serving as the repair template during repair of the cleaved T7 genome. After the transformation of these two plasmids, *E. coli* GB2005 (pBR322-*ampR-cas9-gp10* + pBAC-*cmR-gp10*∗) was pre-induced to express Cas9 nuclease, followed by infection with wild-type T7 phage at a multiplicity of infection (MOI) of 0.001. Upon injection of the T7 genome, the sgRNA-guided Cas9 nuclease specifically cleaved wild-type *gp10*. The resulting double-strand break was repaired using the supplied repair template, generating *gp10*-deficient phages. Primary screening of *gp10*-deficient phages was performed using a double-spot test. The phages forming lysis zones on *E. coli* GB2005 (pBAC-*cmR-gp10*∗) lawns but not on *E. coli* GB2005 lawns were identified as candidate *gp10*-deficient phages and subsequently validated by PCR and Sanger sequencing.

In DCHR, to bypass co-electroporation of large phage genomic DNA with donor templates, a dual-plasmid system was developed to generate linear donor templates (HA*-lox66-gp10-lox71-*HA) in *E. coli* GB2005, followed by infection with T7 (Δ*gp10*) for recombineering (Fig. 1B). The dual-plasmid system comprised two engineered plasmids: a single-copy BAC plasmid (pBAC-*kanR-cas9*-HA) carrying the arabinose-inducible *cas9* gene, and a medium-copy p15A donor plasmid (p15A-*cmR*-HA-*lox66-gp10-lox71*-HA) containing the donor template flanked by two Cas9 nuclease recognition sites (5′- CTGGACCTTATTCCGCGAAG*AGG*-3′). When T7 (Δ*gp10*) infected *E. coli* GB2005 harboring both plasmids, arabinose induction triggered Cas9 expression, followed by CRISPR-Cas9-mediated cleavage of the p15A donor plasmid, thereby releasing the donor template, which contains 500-bp HA flanking a target gene (e.g., *gp4.3–4.7* or other genes of interest), *lox66* and *lox71* sites, and a P_TAC_ promoter-driven *gp10* serving as the selection marker (P_TAC_, a hybrid trp-lacUV5 promoter enabling strong expression in *E. coli* [29]). Then the donor template replaced the target genes (*gp4.3–4.7*) via homologous recombination and was integrated into the T7 (Δ*gp10*) genome. Progeny phages successfully incorporating *gp10* (the selection marker) propagated and formed plaques on *E. coli* GB2005 lawns, whereas the non-recombinant phages lacking *gp10* were unable to replicate. Through this homologous recombination process, gene knockout, insertion, or point mutation can be accomplished. The isolated recombinant phages were subsequently used to infect *E. coli* GB2005 cells carrying the pSC101-BAD-*cre*-*tetR* plasmid (a temperature-sensitive plasmid with an arabinose-inducible promoter driving Cre recombinase expression) [30] and the pBAC-*cmR-gp10*∗ plasmids. Cre recombinase specifically recognized co-directional *lox66* and *lox71* sites, and excised the intervening *gp10*, restoring the *gp10*-deficient genotype. These *gp10*-deficient phages were isolated on *E. coli* GB2005 (pBAC-*cmR-gp10*∗) lawns, validated by PCR, and subjected to additional editing cycles. DCHR thus enabled multiplex genome engineering through an iterative process (Fig. 1B). Finally, upon completion of all editing steps, *gp10* driven by the native T7 promoter was reintroduced downstream of *gp9* via DCHR, thereby restoring its wild-type genotype.

### 2.3 Preparation of phage genomic DNA

Fifty milliliters of log-phase *E. coli* cells (OD_600_ ∼ 0.8) were infected with T7 or T7 mutants at an MOI of 0.001. The culture was incubated at 37 °C for 4–5 h with shaking (200 rpm) until complete lysis occurred. Cellular debris was removed by centrifugation at 8300×*g* for 5 min, and the supernatant was filtered through a 0.22 μm membrane (Millipore, Cat. No. SLGPR33RB). To degrade residual nucleic acids, the clarified lysate was treated with 5 μL of 20 mg mL^−1^ RNase A (Sangon Biotech Co., Ltd., Cat. No. B500474) and 1 μL of 1 mg mL^−1^ DNase I (Sangon Biotech Co., Ltd., Cat. No. A510099) at room temperature for 30 min. Subsequently, 2.92 g NaCl was dissolved in the lysate, followed by incubation at 4 °C for 1 h. Five grams of polyethylene glycol 8000 (PEG 8000) was then introduced, and the solution was incubated overnight at 4 °C. The precipitate was collected by centrifugation at 9500×*g* for 30 min at 4 °C and resuspended in 3 mL of ddH_2_O. For DNA extraction, 500 μL of the resuspended phage solution was mixed with 30 μL of 20 mg mL^−1^ Proteinase K and 40 μL of 10 % (w/v) SDS, followed by incubation at 50 °C for 30 min. Nucleic acids were extracted using 600 μL of the DNA extraction mixture (phenol, chloroform, and isoamyl alcohol, 25:24:1, pH 8.0) and thoroughly mixed by inversion. After phase separation by centrifugation (9500×*g*, 30 min, 4 °C), the upper aqueous layer was transferred to a fresh 2 mL tube. Phage DNA was precipitated with 2.5 vol of ice-cold ethanol. The DNA pellet was washed with 70 % ethanol, air-dried, and resuspended in 400 μL of ddH_2_O.

### 2.4 Overlap extension PCR

Overlap extension PCR consists of several steps designed to seamlessly fuse two or more DNA fragments through complementary overlapping regions introduced by primer design. The primer pairs listed in Table S3 were designed such that the forward primer of the downstream fragment and the reverse primer of the upstream fragment contained complementary overlapping sequences at their 5′ ends. These overlapping sequences enabled the subsequent fusion of DNA fragments. The individual fragments were initially amplified using *Apex*HF HS DNA Polymerase (Accurate Biotechnology Co., Ltd., Cat. No. AG12207) and their respective primer pairs. The PCR products were separated by agarose gel electrophoresis and purified using the TIANgel Purification Kit (TIANGEN, Cat. No. DP219) according to the manufacturer's instructions. After purification to remove excess primers and polymerase, the amplified DNA fragments were mixed in equimolar ratios. During the extension step, the overlapping regions annealed, and *Apex*HF HS DNA Polymerase (Accurate Biotechnology Co., Ltd., Cat. No. AG12207) extended the complementary strands to generate a single fused product. This hybrid DNA then served as the template for the final PCR with the outermost primers, producing a full-length construct containing the joined fragments. The final PCR products were separated by agarose gel electrophoresis and purified using a TIANgel Purification Kit (TIANGEN, Cat. No. DP219) according to the manufacturer's instructions.

### 2.5 Construction of plasmids

The plasmid segments and introduced genes were amplified by PCR or overlap extension PCR using *Apex*HF HS DNA Polymerase (Accurate Biotechnology Co., Ltd., Cat. No. AG12207) and oligonucleotides listed in Table S4. The amplified PCR products were resolved by agarose gel electrophoresis and purified using the TIANgel Purification Kit (TIANGEN, Cat. No. DP219) according to the manufacturer's instructions. The purified DNA was eluted from spin columns with 20 μL ddH_2_O. The target fragments were directionally cloned into BAC, p15A, or pBR322 plasmid backbones through RecET-mediated linear–linear homologous recombination in *E. coli* GB05-dir competent cells [31]. Recombinant colonies were isolated on LB agar plates containing appropriate antibiotics. Putative positive clones were screened using colony PCR and subsequently confirmed by Sanger sequencing.

### 2.6 Construction of deficient T7 phages

The DNA fragments of *cas9*, CRISPR array targeting *gp10*, and repair template were amplified via PCR or overlap extension PCR using *Apex*HF HS DNA Polymerase (Accurate Biotechnology Co., Ltd., Cat. No. AG12207) and oligonucleotides listed in Table S4. These fragments were cloned into the pBR322 plasmid to construct the pBR322-*ampR-cas9*-*gp10* plasmid. The synonymous mutant *gp10* and *gp11* listed in Table S5 were synthesized by Qingdao RuiboXingke Biotechnology Co., Ltd. and cloned with the P_TAC_ promoter into the BAC plasmid to generate pBAC-*cmR-gp10*∗ and pBAC-*cmR-gp11*∗ plasmids.

The construction of the T7 *gp10*-deficient phage was performed as follows: a 40 μL aliquot of overnight culture of *E. coli* GB2005 (harboring both pBAC-*cmR-gp10*∗ and pBR322-*ampR-cas9-gp10* plasmids) was inoculated into 1.4 mL of fresh LB medium containing the appropriate antibiotics and incubated at 30 °C with shaking at 950 rpm for 2 h. Subsequently, 30 μL of 10 % (w/v) arabinose solution was added to induce the expression of Cas9 nuclease, followed by incubation at 37 °C until log-phase growth (OD_600_ = 0.6–0.8, ∼40 min). Wild-type T7 phage was then added at an MOI of 0.001, and incubation continued until complete lysis. The lysate was centrifuged at 9500×*g* to remove cellular debris. The phage-containing supernatant was serially diluted and plated on LB agar with *E. coli* GB2005 (pBAC-*cmR-gp10*∗). After 4–5 h of incubation at 37 °C, individual plaques were isolated using sterile yellow tips and spotted onto *E. coli* GB2005 (pBAC-*cmR-gp10*∗) and *E. coli* GB2005 lawns. Phages forming clear plaques on *E. coli* GB2005 (pBAC-*cmR-gp10*∗) rather than on *E. coli* GB2005 were selected. This screening method was designated as a double-spot test. The knockout of *gp10* was confirmed using PCR and Sanger sequencing.

The construction of the T7 *gp11*-deficient phage followed the same procedure as that of the T7 *gp10*-deficient phage.

### 2.7 Implementation process of DCHR

The DNA fragments of *cas9* and the CRISPR array targeting both ends of the HA-*lox66-gp10-lox71*-HA donor template were amplified via PCR or overlap extension PCR using *Apex*HF HS DNA Polymerase (Accurate Biotechnology Co., Ltd., Cat. No. AG12207) and oligonucleotides listed in Table S4. These fragments were cloned into a BAC plasmid to construct the pBAC-*kanR-cas9*-HA plasmid. The HA-*lox66-gp10-lox71*-HA donor template targeting *gp4.3–4.7* was amplified by overlap extension PCR using the oligonucleotides listed in Table S4 and cloned into the p15A plasmid to generate the p15A-*cmR*-HA-*lox66-gp10-lox71*-HA donor plasmid.

For DCHR, a 40 μL aliquot of overnight *E. coli* GB2005 culture (harboring both pBAC-*kanR-cas9*-HA and p15A-*cmR*-HA-*lox66-gp10-lox71*-HA plasmids) was inoculated into 1.4 mL of fresh LB medium containing the appropriate antibiotics and incubated at 37 °C with shaking at 950 rpm for 2 h until log-phase growth. Moreover, 30 μL of 10 % (w/v) arabinose solution was introduced to induce Cas9 expression, and defective T7 mutants were introduced at an MOI of 0.1. After 40 min of incubation, the culture was centrifuged at 9500×*g* to remove cellular debris. The supernatant was serially diluted, and 100 μL of each dilution was mixed with 100 μL of an overnight *E. coli* GB2005 culture (OD_600_ = 3.0–4.0), incubated at 37 °C for 5 min, combined with 8 mL of molten LB soft agar (0.7 %, w/v), and poured onto LB agar plates. After 4–5 h of incubation at 37 °C, the plaques were counted.

For Cre-lox site-specific recombination, *E. coli* GB2005 carrying the pSC101-BAD-*cre-tetR* plasmid [30] in the log phase was infected with recombinant phages at an MOI of 0.001 and incubated until lysis. The lysate was diluted and plated on *E. coli* GB2005 (pBAC-*cmR-gp10*∗). After 4–5 h at 37 °C, plaques were picked, and the deletion of the selection marker (*gp10*) was verified by colony PCR and Sanger sequencing. The correct recombinant phages were subjected to subsequent rounds of DCHR.

After completing the editing process, *gp10* with the P_T7_ promoter (the native promoter of the T7 phage) was reintroduced downstream of *gp9* via DCHR, thereby restoring the *gp10*-deficient T7 phage to its wild-type genotype.

### 2.8 Implementation process of eDCHR

A 40 μL aliquot of an overnight *E. coli* GB05-red culture was transferred to 1.4 mL of fresh LB medium and incubated at 30 °C with shaking (950 rpm) for 2 h. Subsequently, 30 μL of a 10 % (w/v) arabinose solution was added to induce the expression of Redα and Redβ recombinases. The culture was further incubated at 37 °C until log-phase growth (OD_600_ = 0.6–0.8, ∼40 min). The cells were collected by centrifugation at 9500×*g*, and the pellet was washed twice with 1 mL of ddH_2_O. A total of 5 μg of the purified HA-*lox66-gp10-lox71-*HA donor template was co-electroporated with 20 μg of genomic DNA from defective T7 mutants into *E. coli* GB05-red cells using the Gene Pulser Xcell electroporation system (1800 V, 25 μF, 200 Ω). To ensure consistency in evaluating recombination efficiency, equal amounts of nucleic acids (HA-*lox66-gp10-lox71-*HA) and phage genomic DNA were maintained across all groups. Following electroporation, the cells were immediately resuspended in 1 mL of LB and incubated at 37 °C for 40 min with shaking. The culture was centrifuged at 9500×*g* for 30 s to remove cells. The supernatant containing phages was plated on LB agar seeded with *E. coli* GB2005. Individual plaques were isolated and verified using colony PCR and Sanger sequencing.

For the implementation of eDCHR in *E. coli* GB2005, the addition of arabinose to induce the expression of Redα and Redβ recombinases was unnecessary, whereas all other steps remained identical.

### 2.9 Determination of PFU, recombination efficiency and recombination frequency

After recombination via either the DCHR or eDCHR method, the phage lysate was centrifuged at 8500×*g*, and the supernatant was diluted appropriately. A 100 μL aliquot of the diluted supernatant was mixed with 100 μL of an overnight-cultured host culture (OD_600_ = 3.0–4.0) and incubated at 37 °C for 5 min. The mixture was then combined with 8 mL of molten 0.7 % (w/v) LB soft agar and poured onto LB agar plates. After solidification of the top agar, the plates were inverted and incubated at 37 °C for 4–5 h, after which the number of PFU (plaque-forming units) was counted.

To evaluate recombination efficiency, *E. coli* GB2005 served as the host strain. The PFU on *E. coli* GB2005 lawns was quantified, and the titer of recombinant phages (PFU mL^−1^) was calculated to represent recombination efficiency.

To assess the recombination frequency, both *E. coli* GB2005 and *E. coli* GB2005 (p15A-*cmR-gp10*∗) were used as host strains. The PFU on LB plates for each strain was quantified. The PFU count on *E. coli* GB2005 lawns corresponded to the recombinant PFU, whereas the PFU count on *E. coli* GB2005 (p15A-*cmR-gp10*∗) lawns represented the total PFU. The recombination frequency was calculated as the ratio of recombinant PFU to total PFU.

### 2.10 Plasmid concentration assay

An 80 μL aliquot of an overnight culture of *E. coli* GB2005 (harboring pBAC-*kanR-cas9*-HA and p15A-*cmR*-HA-*lox66-gp10-lox71*-HA plasmids) was transferred into 2.8 mL of LB medium supplemented with kanamycin and chloramphenicol and incubated at 37 °C for 2 h to reach log phase. Different concentrations of arabinose were added, and incubation continued at 37 °C for 10 min. The culture was adjusted to a normalized OD_600_ of 2.0. Plasmid DNA was extracted using the TIANprep Mini Plasmid Kit (TIANGEN, Cat. No. DP103) according to the manufacturer's protocol. The extracted plasmid DNA was digested with the restriction endonuclease *Apa*LI for linearization. Ten μL of the digested products was separated by agarose gel electrophoresis, and the gel electrophoresis image was analyzed using ImageJ software to quantify DNA bands.

### 2.11 β-Galactosidase activity assay

T7 phage mutants carrying the *lacZ* gene (Table S5) were inoculated into 1 mL of log-phase *E. coli* GB2005 culture at an MOI of 0.001. The mixture was incubated at 37 °C with shaking at 950 rpm until the culture became clear. β-Galactosidase activity was quantified using the β-Galactosidase Activity Assay Kit (Sangon Biotech Co., Ltd., Cat. No. D799423) according to the manufacturer's instructions.

### 2.12 Measurement of *E. coli* growth curves

The overnight *E. coli* culture was adjusted to an OD_600_ of 3.0 and inoculated at 6 % (v/v) inoculum into 1.8 mL LB medium with ampicillin and chloramphenicol. The cultures were grown at 37 °C with shaking at 200 rpm, and OD_600_ values were recorded every 30 min.

### 2.13 Statistical analysis

Statistical comparisons between two groups were performed using a two-sided Student's *t*-test. Significance levels are indicated as follows: ∗*P* < 0.05, ∗∗*P* < 0.01, ∗∗∗*P* < 0.001, and ns, not significant. Error bars represent standard deviation (S.D.).

## 3 Results

### 3.1 Phage essential genes used as the selection marker of DCHR are universal

Phage essential genes encode functions indispensable for infection progression, including genome replication, virion assembly, and host lysis. Disruption of these genes can abolish phage propagation. In this study, the T7 essential genes *gp10* (Gene ID: 1261026, 1.04 kb) and *gp11* (Gene ID: 1261030, 0.59 kb), which encode the capsid protein and tail tubular protein, respectively [32], were selected as the selection marker genes for DCHR. Following the procedure described in Section 2.2, DCHR was employed to achieve the targeted knockout of the non-essential T7 genes *gp4.3–4.7*.

**Fig. 2:**
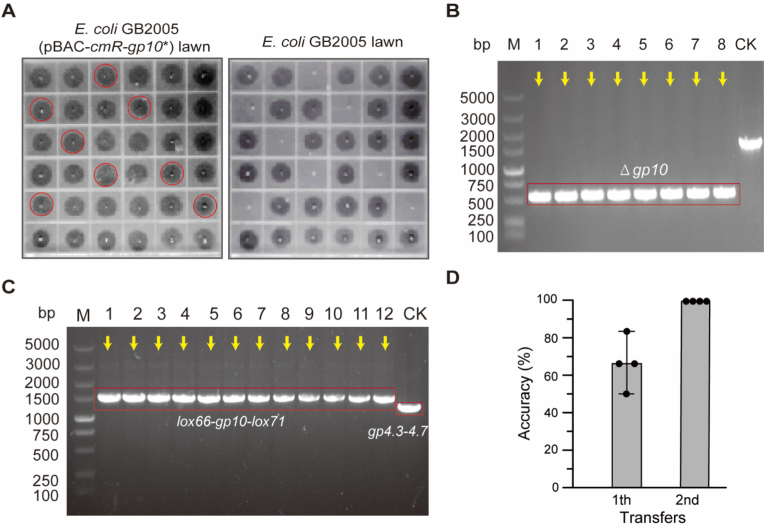
Deletion of *gp4.3–4.7* via DCHR. (A) Screening of T7 *gp10*-deficient phages using a double-spot test. The eight candidate phages are highlighted in red. (B) PCR verification of *gp10* deletion. The correct phages are indicated by yellow arrows. Wild-type T7 phage was used as the control (CK). (C) PCR verification of recombinant phages. Twelve randomly selected recombinant T7 phages were analyzed by colony PCR. Correctly recombinant phages are indicated by yellow arrows. Wild-type T7 phage was used as the control (CK). (D) Evaluation of Cre-lox iterative recombination accuracy. *n* = 4 independent experiments. Data are presented as mean ± standard deviation (S.D.).

Initially, *gp10* was adopted as the selection marker, and CRISPR-Cas9 technology was applied to generate a *gp10*-deficient T7 phage (Fig. 1A). Phages were recovered from *E. coli* GB2005 (pBAC-*cmR-gp10*∗) lawns, purified, and subsequently spotted onto two types of *E. coli* lawns: one harboring the pBAC-*cmR-gp10*∗ plasmid and the other lacking it. Thirty-six randomly selected phages were subjected to double-spot testing. The results demonstrated that eight phages produced lysis zones on *E. coli* GB2005 (pBAC-*cmR-gp10*∗) lawns but failed to lyse *E. coli* GB2005 lawns (Fig. 2A). Subsequent PCR testing followed by Sanger sequencing confirmed the absence of *gp10* in all eight phage genomes (Fig. 2B).

Overlap extension PCR was used to construct the donor template (HA-*lox66-gp10-lox71*-HA) with 500-bp HA flanking the *gp4.3–4.7*. The donor template was cloned into the p15A donor plasmid and subsequently transferred into the *E. coli* GB2005 (pBAC-*kanR-cas9*-HA). When T7 (Δ*gp10*) infected this recombinant *E. coli* GB2005 at an MOI of 0.1, arabinose (2 g L^−1^) was introduced to induce Cas9 expression. After 40 min of incubation, phages were isolated on *E. coli* GB2005 lawns, and twelve recombinant T7 phages were randomly selected for PCR analysis. The 1.02-kb *gp4.3*–*4.7* was completely replaced by the 1.21-kb *lox66-gp10-lox71* cassette, which was further validated by Sanger sequencing (Fig. 2C). These results demonstrated that the recombination accuracy of DCHR using *gp10* as the selection marker reached 100 % (12/12).

The Cre site-specific recombinase specifically recognizes the co-directional *lox66* and *lox71* sites and excises the intervening *gp10* [33,34]. Following induction with 2 g L^−1^ arabinose to express Cre recombinase in *E. coli* GB2005 (pSC101-BAD-*cre-tetR* + pBAC-*cmR-gp10*∗), the culture was infected with recombinant T7 (Δ*gp10*, *gp4.3*–*gp4.7::lox66-gp10-lox71*) phage at an MOI of 0.001. After complete lysis, phages were isolated from *E. coli* GB2005 (pBAC-*cmR-gp10*∗) lawns. Six recombinant phages were analyzed using PCR to assess Cre-lox recombination accuracy. In the first round, the *lox66-gp10-lox71* cassette was excised in 66.7 % of the phage genomes (4/6), whereas in the second round, complete excision was achieved in all cases (6/6, 100 % accuracy). These results demonstrated that Cre-lox site-specific recombination enabled the efficient excision of the *lox66-gp10-lox71* cassette (Fig. 2D and S1). At this stage, *gp4.3–4.7* was successfully knocked out of the T7 genome.

The same strategy was applied to construct a *gp11*-deficient T7 phage, with *gp11* serving as the selection marker for the knockout of *gp4.3-4.7* via DCHR. Four candidate T7 mutants with *gp11* deficiency were identified using a double-spot test (Fig. S2). PCR analysis and Sanger sequencing confirmed the successful knockout of *gp11* in all four T7 mutants (Fig. S3). Upon infecting *E. coli* GB2005 (harboring p15A-*cmR*-HA-*lox66-gp11-lox71*-HA and pBAC-*kanR-cas9*-HA plasmids) with T7 (Δ*gp11*), twelve recombinant phages were randomly selected from plaques on *E. coli* GB2005 lawns for PCR validation. The *gp4.3*–*4.7* was completely replaced by the *lox66-gp11-lox71* cassette (Fig. S4). These results demonstrated that DCHR using *gp11* as the selection marker also achieved 100 % recombination accuracy (12/12).

During T7 phage particle assembly, Gp10 (415 copies per virion) and Gp11 (∼6 copies per virion) exhibit a marked stoichiometric disparity [32,35]. The expression of engineered essential genes potentially interferes with T7 phage growth. In this work, both *gp10* and *gp11* were successfully employed as selection markers for DCHR, indicating that their expression levels were sufficient to maintain the normal growth of recombinant T7 phages. Despite a marked stoichiometric disparity between the encoded proteins, their functional equivalence (both achieving 100 % accuracy) as selection markers suggests that phage essential genes may universally serve as DCHR selection markers.

### 3.2 DCHR exhibits high recombination efficiency independent of exogenous homologous recombination systems and electroporation of phage genomic DNA

The reliance on exogenous homologous recombination systems and electroporation for phage genomic DNA delivery limits the applicability of existing phage genome-editing tools. Numerous studies have confirmed that extending HA length facilitates homologous recombination efficiency [36,37]. In this study, we systematically evaluated the effect of HA length on the DCHR recombination efficiency, aiming to achieve high recombination efficiency without exogenous homologous recombination systems (the λ-Red recombination system [38]) and electroporation of phage genomic DNA.

**Fig. 3:**
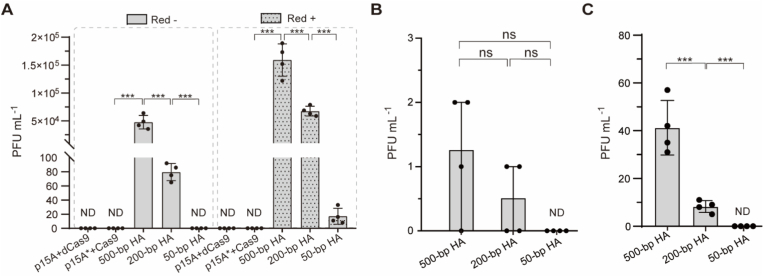
Determination of DCHR and eDCHR recombination efficiencies. (A) Comparison of recombination efficiency between DCHR without (Red-) and with (Red+) the λ-Red recombination system. The p15A∗ plasmid denotes the p15A donor plasmid without Cas9 nuclease recognition sites flanking both ends of the donor template. (B) Recombination efficiency of eDCHR in the absence of the λ-Red recombination system. (C) Recombination efficiency of eDCHR in the presence of the λ-Red recombination system. *n* = 4 independent experiments. Data are presented as mean ± S.D. Statistical analysis between two groups was performed using a two-sided Student's *t-*test. ∗*P* < 0.05; ∗∗*P* < 0.01; ∗∗∗*P* < 0.001; ns, not significant. ND, no recombinant phages were detected.

Using overlap extension PCR, a series of HA sequences (500, 200, and 50 bp) flanking *gp4.3*–*4.7* were ligated to both ends of the *lox66-gp10-lox71* cassette, cloned into the p15A donor plasmids, and transformed into *E. coli* GB2005 harboring the pBAC-*kanR-cas9*-HA plasmid. DCHR was conducted in the recombinant *E. coli* GB2005 lacking the λ-Red recombination system in its chromosome. The obtained phage mixtures were plated on *E. coli* GB2005 lawns, incubated at 37 °C for 4 h, and the PFU were quantified. With 500-bp HA, the recombinant phage titer reached (4.58 ± 1.35) × 10^4^ PFU mL^−1^. This value decreased substantially to 79 ± 13 PFU mL^−1^ with 200-bp HA, whereas no recombinant phages were identified with 50-bp HA. Parallel experiments in *E. coli* GB05-red with the chromosomal λ-Red recombination system demonstrated higher recombination efficiency. The maximal yield (1.50 ± 0.33) × 10^5^ PFU mL^−1^ was achieved with 500-bp HA, decreasing to (6.53 ± 0.90) × 10^4^ PFU mL^−1^ with 200-bp HA, and minimal production (15 ± 8) PFU mL^−1^ with 50-bp HA (Fig. 3A). These results indicated that the absence of the λ-Red recombination system led to a nearly three-orders-of-magnitude decrease in recombinant phage titer with 200-bp HA. However, long HA (≥500 bp) compensated for this deficiency and also achieved a substantial titer of (4.58 ± 1.35) × 10^4^ PFU mL^−1^. Since 500-bp HA produced sufficient recombinant phage titer for isolation, no further extension of HA was performed in DCHR. Furthermore, no recombinant phages were produced in either the recombinant *E. coli* GB2005 or *E. coli* GB05-red strains under the following conditions: (i) replacement of the wild-type *cas9* gene in the BAC plasmid with its catalytically inactive variant dCas9 (D10A/H840A), or (ii) use of the p15A donor plasmid lacking Cas9 nuclease recognition sites (designated p15A∗ plasmid) (Fig. 3A). These findings suggest that the linear donor template released through Cas9-mediated cleavage of p15A donor plasmids plays a critical role in DCHR. The failure of the circular donor template in the p15A donor plasmid to generate recombinant phages may result from selective recognition of exposed DNA ends by the RecBCD complex, a crucial enzyme complex involved in homologous recombination [39,40].

Electroporation has been widely used to deliver phage genomic DNA in phage genome engineering [15,18,41]. However, the efficiency of electroporation for large phage genomic DNA delivery remains limited, particularly in strains with low transformation efficiency [41]. To avoid this constraint, DCHR employs two delivery strategies: (i) intracellular release of donor templates via CRISPR-Cas9 cleavage of p15A donor plasmids, and (ii) direct delivery of phage genomic DNA through phage infection (Fig. 1B). To assess the impact of different delivery methods on recombination efficiency, DCHR was compared with the electroporation-based DCHR (eDCHR), where both donor templates and phage genomic DNA were introduced by electroporation (Fig. S5). Using overlap extension PCR, HA sequences targeting *gp4.3*–*4.7* were fused to the *lox66-gp10-lox71* cassette. These donor templates were co-electroporated with T7 (Δ*gp10*) genomic DNA into *E. coli* GB2005 and *E. coli* GB05-red. The resulting phage mixtures were plated on *E. coli* GB2005 lawns, incubated at 37 °C for 4 h, and the PFU were counted. When eDCHR was performed in *E. coli* GB2005, the recombination efficiency was significantly reduced. The 500-bp HA generated a recombinant phage titer of 1.3 ± 0.8 PFU mL^−1^, the 200-bp HA yielded 0.5 ± 0.5 PFU mL^−1^, and no plaques were observed with 50-bp HA (Fig. 3B). In *E. coli* GB05-red, the 500-bp HA produced the highest recombinant phage titer of 41 ± 10 PFU mL^−1^, whereas the 200-bp HA yielded only 8 ± 2 PFU mL^−1^, and no recombinants were detected with 50-bp HA (Fig. 3C). These findings clearly indicated that in *E. coli* GB05-red as a strain derived from the high-electroporation-efficiency *E. coli* DH10B [42], the recombination efficiency of eDCHR was relatively lower than that of DCHR. This disparity was more pronounced in *E. coli* MG1655 (a low-electroporation-efficiency strain [43]), where eDCHR failed to generate recombinant phages with and without the λ-Red recombination system. In contrast, DCHR achieved a recombinant phage titer of (1.15 ± 0.13) × 10^4^ PFU mL^−1^ in *E. coli* MG1655 with 100 % accuracy (12/12) (Fig. S6). These results indicate that the delivery strategy for phage genomic DNA in DCHR can substantially enhance the recombination efficiency. This enhancement may be attributed to the increased opportunity for phage genomic DNA and donor templates to engage in recombineering processes.

### 3.3 Optimizations of Cas9 expression to enhance DCHR recombination efficiency

In DCHR, we observed that the circular donor template (HA*-lox66-gp10-lox71-*HA) within the p15A donor plasmid hindered the generation of recombinant phages, indicating that the intracellular release of linear donor template was crucial (Fig. 3A). To enhance DCHR recombination efficiency, both the induction timing and expression level of Cas9 nuclease were optimized. The recombination frequency, defined as the ratio of recombinant phage titer to total phage titer, was used as the evaluation metric for these optimization strategies.

**Fig. 4:**
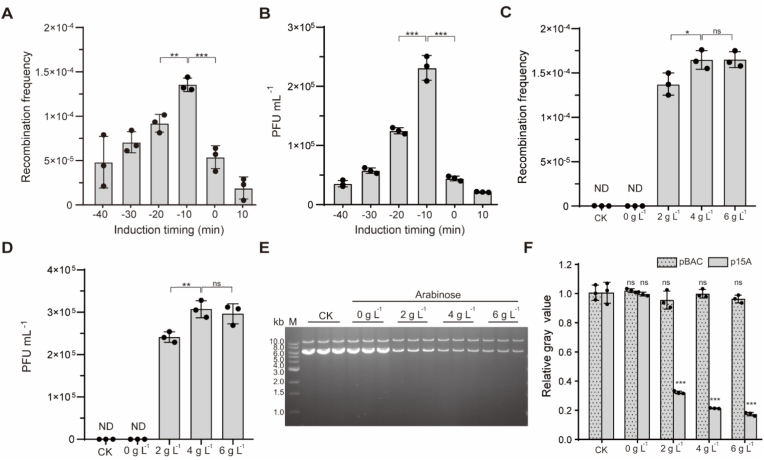
Enhancement of DCHR recombination efficiency through Cas9 expression optimization. (A) Effect of Cas9 nuclease induction timing on the recombination frequency. (B) Effect of Cas9 nuclease induction timing on the recombination efficiency. (C) Effect of arabinose concentrations on the recombination frequency. (D) Effect of arabinose concentrations on the recombination efficiency. (E) Detection of plasmid concentrations using agarose gel electrophoresis. Plasmid DNA was linearized with *Apa*LI restriction endonuclease and separated by agarose gel electrophoresis. (F) Relative gray values of the plasmid DNA bands in panel E. Gray values were calculated using ImageJ software. *E. coli* GB2005 harboring the p15A∗ plasmid (lacking Cas9 nuclease recognition sites) and pBAC-*kanR-Cas9*-HA plasmid was used as the control (CK). *n* = 3 independent experiments. Data are presented as mean ± S.D. Statistical analysis comparing the experimental and control groups was performed using a two-sided Student's *t-*test. ∗*P* < 0.05; ∗∗*P* < 0.01; ∗∗∗*P* < 0.001; ns, not significant. ND, no recombinant phages were detected.

To examine the influence of Cas9 induction timing on recombination efficiency of DCHR, T7(Δ*gp10*) was added at different time points after arabinose-induced Cas9 expression at an MOI of 0.1. Lysates were centrifuged at 9500×*g*, and equal volumes of the supernatant were plated on *E. coli* GB2005 and *E. coli* GB2005 (p15A-*cmR-gp10*∗) lawns. The PFU was quantified, and the recombination frequency was determined. The results indicated that preinduction of Cas9 significantly enhanced recombination frequency. The induction at 10 min before T7 (Δ*gp10*) infection (designated as −10 min) produced a maximum recombination frequency of (1.36 ± 0.14) × 10^−4^, which was 2.6-fold higher than that of simultaneous induction and infection (0 min) and 1.5-fold higher than that of induction at 20 min before infection (−20 min) (Fig. 4A). Under this optimal condition (−10 min), the recombinant phage titer reached (2.31 ± 0.21) × 10^5^ PFU mL^−1^, representing a 5.1-fold enhancement over that of the 0 min induction and 1.8-fold increase over that of the −20 min induction (Fig. 4B).

Subsequent attempts involved in optimizing arabinose induction concentration and plasmid copy number were conducted to enhance Cas9 nuclease expression levels for improving recombination efficiency. With 4 g L^−1^ arabinose, recombination frequency peaked at (1.65 ± 0.11) × 10^−4^, which was 1.3-fold increase compared to 2 g L^−1^ arabinose (Fig. 4C). Similarly, recombination efficiency at 4 g L^−1^ arabinose reached the maximum of (3.07 ± 0.20) × 10^5^ PFU mL^−1^, which was 1.3-fold higher than at 2 g L^−1^ arabinose (Fig. 4D). When the arabinose concentration exceeded 4 g L^−1^, neither recombination frequency nor recombination efficiency showed further improvement. No recombinant phages were detectable under non-induced conditions or upon replacement of the p15A plasmid with the p15A∗ plasmid at 4 g L^−1^ arabinose (the control group).

Plasmid DNA concentrations during arabinose-induced Cas9 expression were quantified via agarose gel electrophoresis. Following adjustment of OD_600_ to 2.0 post-induction, *E. coli* cells (harboring the pBAC-*kanR-Cas9*-HA and p15A-*cmR-*HA-*lox66-gp10-lox71*-HA plasmids) were harvested for plasmid extraction. Plasmid DNA was separated by agarose gel electrophoresis and quantified based on DNA band gray values (Fig. 4E). The 13.4-kb pBAC-*kanR-Cas9*-HA plasmid maintained stable concentrations regardless of arabinose induction, while the 6.4-kb p15A donor plasmid displayed arabinose-dose-dependent degradation (Fig. 4F). Compared to non-induced conditions (0 g L^−1^), relative gray values of the p15A donor plasmid were reduced to 30 %, 21 %, and 19 % at 2, 4, and 6 g L^−1^ arabinose, respectively. The concentration of the p15A∗ plasmid (the control group) remained unaffected by arabinose-induced Cas9 expression (4 g L^−1^), with comparable levels observed under the non-induced conditions (Fig. 4F). This arabinose-dose-dependent degradation of the p15A donor plasmid confirmed specific Cas9-mediated cleavage.

Subsequent engineering involved cloning *cas9* into the medium-to-high copy number pBR322 plasmid. However, the *E. coli* GB2005 strain carrying both pBR322-*ampR*-*cas9*-HA and p15A donor plasmids exhibited significant growth retardation in LB medium with ampicillin and chloramphenicol (Fig. S7). We hypothesized that the observed growth impairment could result from Cas9 leaky expression driven by elevated plasmid copy numbers, which triggered p15A donor plasmid degradation and impaired cellular proliferation. These findings suggest that employing a single-copy BAC plasmid for Cas9 expression may be the optimal strategy.

### 3.4 Generation of gene deletion and insertion via DCHR

**Fig. 5:**
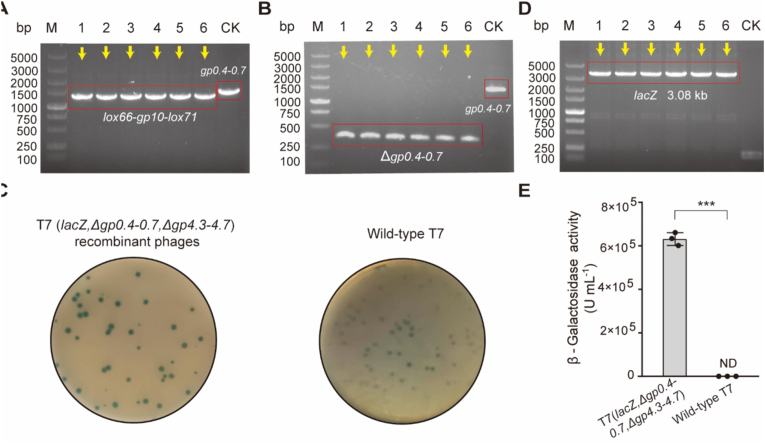
Generation of gene deletion and insertion via DCHR. (A) Verification of recombination accuracy using PCR. Six recombinant T7 phages were randomly selected and analyzed using colony PCR. Wild-type T7 phage was used as the control (CK). The correct recombinant phages are indicated by yellow arrows. (B) Verification of Cre-lox recombination accuracy by PCR. Six recombinant T7 phages were randomly selected and analyzed using colony PCR. Correct recombinant phages are indicated by yellow arrows. Wild-type T7 phage was used as the control (CK). (C) Expression of *lacZ* in recombinant phages. On X-gal-containing *E. coli* GB2005 lawns, all plaques for recombinant phages appeared blue, whereas those of wild-type T7 remained colorless. (D) PCR verification of *lacZ* insertion. Correct recombinant phages carrying *lacZ* are indicated by yellow arrows. Wild-type T7 phage was used as the control (CK). (E) Determination of β-galactosidase activity in recombinant phages harboring *lacZ*. *n* = 3 independent experiments. Data are presented as mean ± S.D. Statistical analysis between two groups was performed using a two-sided Student's *t*-test. ∗*P* < 0.05; ∗∗*P* < 0.01; ∗∗∗*P* < 0.001; ns, not significant. ND, no β-galactosidase activity was detected.

To validate DCHR as a phage genome-editing tool, additional genetic manipulations were performed to delete another 1.48-kb nonessential genomic region (*gp0.4–0.7*) in the T7(Δ*gp10*, Δ*gp4.3-4.7*) genome. Using overlap extension PCR, the 500-bp HA sequences flanking *gp0.4–0.7* were fused into the *lox66-gp10-lox71* cassette and cloned into the p15A donor plasmid in *E. coli* GB2005 harboring the pBAC-*kanR-Cas9*-HA plasmid. The implementation of the DCHR protocol resulted in complete replacement of the 1.48-kb *gp0.4*–*0.7* region with the 1.21-kb *lox66-gp10-lox71* cassette in six randomly selected recombinant phages (Fig. 5A). The *gp10* selection marker was subsequently excised using Cre-lox site-specific recombination, as confirmed by PCR and Sanger sequencing (Fig. 5B).

DCHR was subsequently employed for gene insertion. Because the limited packaging capacity of the wild-type T7 genome could restrict insertions beyond ∼3 kb [15], the successive deletion of *gp10* (1.04 kb), *gp4.3*–*4.7* (1.02 kb), and *gp0.4–0.7* (1.48 kb) generated a T7 mutant with a 3.54-kb genome reduction and this reduction enabled the insertion of a 4.12-kb *gp10-lacZ* fragment (3.08 kb *lacZ*, encoding β-galactosidase, a widely used reporter gene) downstream of *gp9*. The donor template, consisting of the *gp10-lacZ* fragment and 500-bp HA flanking *gp10*, was constructed by overlap extension PCR, cloned into the p15A donor plasmid and transformed into *E. coli* GB2005 harboring the pBAC-*kanR-Cas9*-HA plasmid. Following DCHR-mediated editing, a recombinant T7 (*lacZ*) phage titer of (2.88 ± 0.37) × 10^4^ PFU mL^−1^ was obtained (Fig. S8). Plaques on *E. coli* GB2005 lawns with X-gal displayed uniform blue coloration, whereas wild-type T7 plaques remained colorless, confirming functional *lacZ* expression in all recombinant phages (Fig. 5C). PCR analysis of six randomly selected blue plaques validated *lacZ* integration (Fig. 5D). Quantitative assays further demonstrated substantial β-galactosidase activity (6.31 ± 0.29) × 10^5^ U mL^−1^ in the recombinant phage lysates, whereas no activity was detected in wild-type T7 phage lysates (Fig. 5E). These results confirmed the successful genomic integration and functional expression of the *lacZ* reporter gene. In addition, the genetic stability of recombinant T7 (*lacZ*) phage was evaluated by performing 10 transfers (60–80 generations) in the host *E. coli* GB2005 at an MOI of 0.001. The *lacZ* gene was confirmed by PCR in six progeny phage genomes after 10 transfers, demonstrating stable maintenance of the *lacZ* gene after 10 transfers (Fig. S9). Furthermore, β-galactosidase activity persisted in the six progeny phage lysates (Fig. S10), indicating that the *lacZ* gene remained fully functional.

### 3.5 Point mutation via DCHR

**Fig. 6:**
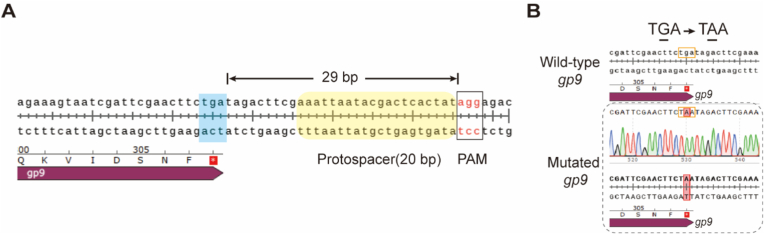
Point mutation of the *gp9* stop codon via DCHR. (A) Screening of the PAM sequence near the *gp9* stop codon. The closest PAM sequence to the *gp9* stop codon is located 29 bp downstream. (B) Mutation of the *gp9* stop codon from TGA to TAA via DCHR. Wild-type and mutant *gp9* sequences were aligned using SnapGene software. The point mutation base pair is highlighted in red.

CRISPR-Cas9 gene-editing technology has been extensively applied to phage genome modification. However, its utility remains constrained by protospacer adjacent motif (PAM) sequences [24]. This limitation is particularly evident when introducing point mutations at loci distal to PAM sites, such as the stop codon of *gp9* in the T7 genome. Using the CRISPOR tool (https://crispor.gi.ucsc.edu/crispor.py) [44], the nearest useable PAM sequence was identified 29 bp downstream of the *gp9* stop codon. Because the <20 bp operational window of CRISPR-Cas9 systems upstream of PAM motifs was exceeded, this 29-bp distance precluded Cas9-mediated editing at the target site (Fig. 6A).

In contrast, the DCHR genome-editing technology relies on DNA homologous recombination and can theoretically modify any genomic locus. To demonstrate the editing flexibility of DCHR, a point mutation converting the *gp9* stop codon from TGA to TAA was introduced. The procedure involved (i) designing mutation-containing primers, (ii) amplifying 500-bp HA sequences flanking the *gp9* mutation site, and (iii) assembling these fragments with *gp10* via overlap extension PCR to construct the donor template. The resulting donor template was cloned into the p15A donor plasmid and transformed into *E. coli* GB2005 harboring the pBAC-*kanR-Cas9*-HA plasmid. Following DCHR implementation, recombinant phages with the TGA-to-TAA mutation were successfully obtained and validated by Sanger sequencing (Fig. 6B).

## 4 Discussion

Synthetic biology has emerged as a major driver in the development of phage therapy by enabling systematic genomic reprogramming of phages [7,8,45]. Through synthetic biology approaches, phage-host interactions can be rationally redesigned, thereby broadening host range and enhancing antibacterial efficacy [7,11,46]. This methodology is grounded in three foundational components: (i) Genomic decoding, which provides a comprehensive analysis of structural and functional architectures within phage genomes; (ii) Phenotypic predictability, involving the establishment of genotype-phenotype correlations; and (iii) Precise editing, which entails the development of efficient genome engineering tools with single-nucleotide resolution to restructure phage genomes. Within this framework, precise and efficient genome-editing technologies can accelerate both the structural characterization of genomes and the elucidation of genotype-phenotype correlations, thereby advancing the application of synthetic biology in phage therapeutic development.

Homologous recombination is a critical DNA repair mechanism that uses sequence homology to repair double-strand breaks during replication, thereby maintaining genomic stability. In gene-editing technology, homologous recombination strategies facilitate precise gene insertion, deletion, and site-specific substitution. Owing to its high precision and broad applicability, homologous recombination has been widely applied in model organism engineering, crop improvement, and microbial metabolic engineering [[47], [48], [49]]. However, its application in gene editing remains fundamentally constrained by low natural recombination frequency (10^−4^ to 10^−8^) [16,17], which demonstrates the demand for antibiotic resistance or other dominant selection markers for efficient screening. This limitation is particularly evident during the genome modification of virulent phages, as their rapid lytic cycle involves immediate host cell lysis, rendering conventional antibiotic-based selection systems ineffective.

The DCHR system established in this study addressed the major limitation of homologous recombination in virulent phage genome editing through the innovative application of phage essential genes as selection markers. The advantages of DCHR are as follows. (i) Exceptional accuracy (100 %): Only progeny phages that successfully undergo homologous recombination acquire the essential gene, enabling plaque formation on wild-type host lawns and eliminating laborious recombinant phage isolation. (ii) High fidelity. As a homologous recombination-based gene-editing technology, the DCHR system achieved precise genomic modifications in the T7 genome. Whole-genome sequencing in this study revealed no detectable off-target mutations in non-targeted genome regions of the T7 genome. The whole-genome sequencing data (GSA: Accession No. CRA031453) and assembled genome (GenBase: Accession No. C_AA120783.1) of the recombinant T7 phage with *lacZ* have been deposited in the National Genomics Data Center. (iii) Operational simplicity. The protocol requires only the construction of a plasmid containing homology arms, *lox66*/*lox71* sites, and a selection marker, with each editing cycle completed within approximately 72 h. (iv) Iterative genome-editing capability: Cre-lox-mediated excision of the selection marker enables repeated genome-editing operations at multiple loci. (v) Broad applicability: DCHR achieves high recombination efficiency [(3.07 ± 0.20) × 10^5^ PFU mL^−1^] without relying on exogenous recombination systems or electroporation for phage genomic DNA delivery, allowing its application in the genetic modification of diverse phages. Collectively, these features indicate that DCHR has substantial potential as a general-purpose phage genome-editing tool.

Phage discovery has been ongoing for over a century, but our understanding of phage genomic architecture remains limited. A substantial proportion of open reading frames remains functionally uncharacterized. For instance, in the T4 phage, 128 out of 278 identified genes (46 %) currently lack definitive functional annotations [50]. This annotation gap is significant. A large-scale metagenomic analysis performed by Nikos C. Kyrpides' group, which processed 5 TB of global sequencing data, revealed that more than 75 % of the 2.7 million viral protein sequences currently available remain without precise functional annotations [51]. Such widespread genetic obscurity may introduce potential risks to phage therapy. Within this context, the DCHR system is expected to provide a reliable technical framework for elucidating the functions of uncharacterized genes, enabling accurate genotype-phenotype mapping, and clarifying phage-host interactions.

## 5 Conclusions

The lack of effective selection markers is a critical bottleneck in virulent phage genome editing. This study proposed an innovative strategy that employed phage essential genes (*gp10* and *gp11*) as selection markers. We demonstrated that these genes enabled 100 % recombination accuracy, significantly streamlining the otherwise labor-intensive process of recombinant phage isolation. Moreover, we optimized HA length and Cas9 expression. These improvements allowed DCHR to achieve a high recombination efficiency (3.1 × 10^5^ PFU mL^−1^), even in the absence of exogenous homologous recombination systems. Collectively, these findings suggest that DCHR has the potential to become a precise and efficient general phage genome-editing tool.

## CRediT authorship contribution statement

**Hailin Zhang:** Writing – original draft, Methodology, Formal analysis. **Yueyue Song:** Investigation, Data curation. **Wenyue Liu:** Formal analysis, Data curation. **Xiaoqing Zheng:** Data curation, Conceptualization. **Xiaodong An:** Resources, Methodology. **Chao Li:** Resources, Methodology. **Weihua Chen:** Writing – review & editing. **Hailong Wang:** Writing – review & editing. **Yuran Zhang:** Writing – review & editing.

## Funding

None.

## Declarations of interest

The authors declare that they have no known competing financial interests or personal relationships that could have appeared to influence the work reported in this paper.

## Data Availability

The assembled genome of the recombinant T7 phage with *lacZ* has been deposited in the GenBase of National Genomics Data Center (https://ngdc.cncb.ac.cn/genbase. Accession No. C_AA120783.1). The raw data of whole-genome sequencing for the recombinant T7 phage with lacZ have been deposited in the Genome Sequence Archive of National Genomics Data Center (https://ngdc.cncb.ac.cn/gsa. Accession No. CRA031453).
