# Supplementary material for: Defect-complementation homologous recombination: A novel strategy for precise genome engineering of virulent phages
Source: Synth Syst Biotechnol. 2025 Nov 17;12:59–70. doi: 10.1016/j.synbio.2025.11.002 (PMC12664982; doi:10.1016/j.synbio.2025.11.002)
Supplement: Multimedia component 1 [file mmc1.docx]

**Supplementary Information**

**Defect-****Complementation Homologous Recombination: A Novel Strategy for Precise Genome Engineering of Virulent Phages**

Hailin Zhang^1,4^, Yueyue Song^1^, Wenyue Liu^1^, Xiaoqing Zheng^1^, Xiaodong An^2^, Chao Li^2^, Weihua Chen^1,3^, Hailong Wang^4,5^ & Yuran Zhang^1*^

^1^ School of Life Sciences, Jining Medical University, No. 669 Xueyuan Road, Donggang District, Rizhao, Shandong Province, 276826, China

^2^ Rizhao Center for Disease Control and Prevention, No. 136, Beijing Road, Donggang District, Rizhao, Shandong Province, 276826, China

^3^ Key Laboratory of Molecular Biophysics of the Ministry of Education, Hubei Key Laboratory of Bioinformatics and Molecular Imaging, Center for Artificial Intelligence Biology, Department of Bioinformatics and Systems Biology, College of Life Science and Technology, Huazhong University of Science and Technology, Wuhan, Hubei, China

^4^ State Key Laboratory of Microbial Technology, Institute of Microbial Technology, Helmholtz International Lab for Anti-infectives, Shandong University–Helmholtz Institute of Biotechnology, Shandong University, Qingdao, Shandong, 266237, China

^5^ Rizhao Research Institute of Shandong University, Rizhao, Shandong, 276800, China

Hailin Zhang: happyzhlin@163.com

Yueyue Song: 2821537576@qq.com

Wenyue Liu: liu13375540016@163.com

Xiaoqing Zheng: 3175972603@qq.com

Xiaodong An: an06332281@163.com

Chao Li: cdclicao@163.com

Weihua Chen: weihuachen@hust.edu.cn

Hailong Wang: wanghailong@sdu.edu.cn

Yuran Zhang: todrzhang666@mail.jnmc.edu.cn

Corresponding authors: Weihua Chen: [weihuachen@hust.edu.cn](mailto:weihuachen@hust.edu.cn); Hailong Wang. Email: [wanghailong@sdu.edu.cn](mailto:wanghailong@sdu.edu.cn); Yuran Zhang. Email: todrzhang666@mail.jnmc.edu.cn

**Fig. S1.** PCR analysis of the *lox66-gp10-lox71* fragment excision during iterative Cre-lox site-specific recombination.

**Fig. S2.** Isolation of *gp11*-deficient T7 phages using double-spot test.

**Fig. S3.** PCR analysis of *gp11* deletion.

**Fig. S4.** Verification of recombination accuracy with *gp11* as the selection marker by PCR.

**Fig. S5.** Schematic diagram of eDCHR.

**Fig. S6.** Recombination efficiency of DCHR and eDCHR in *E. coli* MG1655.

**Fig. S7.** Growth curves of *E. coli* GB2005 carrying the pBR322-ampR-cas9-HA and p15A-cmR-HA-lox66-gp10-lox71-HA plasmids.

**Fig. S8.** Recombination efficiency of DCHR in inserting the *lacZ* gene.

**Fig. S9.** PCR amplification of the *lacZ* gene in the genomes of recombinant T7 (*lacZ*) progeny phages.

**Fig. S10.** Determination of β-galactosidase activity in six recombinant T7 (*lacZ*) progeny phages after 10 transfers.

**Table S1.** Nonessential gene list in T7 phage genome.

**Table S2.** Bacteria and phages used in this work.

**Table S3.** Oligonucleotides for construction of cassettes.

**Table S4.** Oligonucleotides for construction of plasmids

**Table S5.** List of gene sequences.


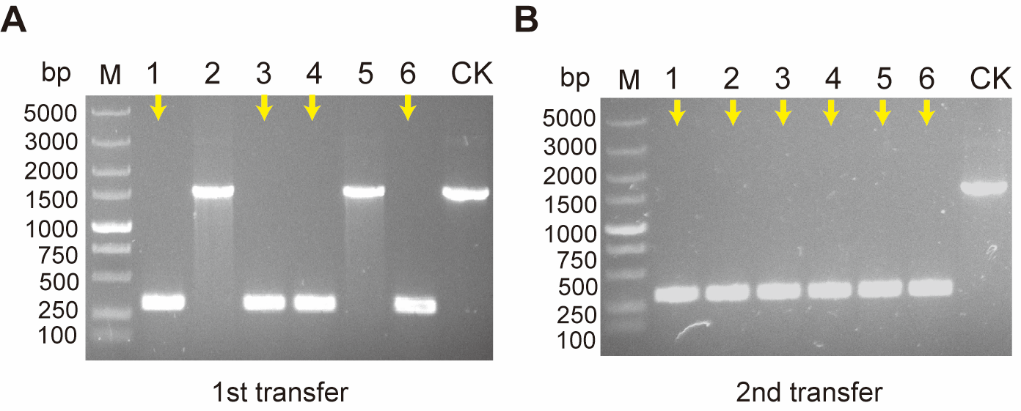


**Fig. S1.** PCR analysis of the *lox66-gp10-lox71* cassette excision during iterative Cre-lox site-specific recombination. (A) PCR analysis of first-round Cre-lox site-specific recombination. (B) PCR analysis of second-round Cre-lox site-specific recombination. Six recombinant phages were randomly selected for PCR verification of the *lox66-gp10-lox71* cassette excision. The correct phages are indicated by yellow arrows. The T7 phage (Δ*gp10*, *gp4.3-4.7::lox66-gp10-lox71*) was used as the control (CK).


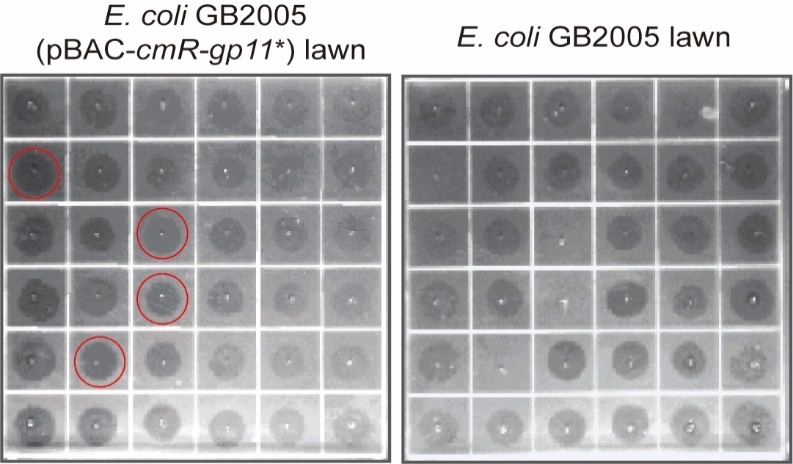


**Fig. S2.** Isolation of *gp11*-deficient T7 phages using double-spot test. The four candidate *gp11*-deficient T7 phages were highlighted in red. The pBAC-*cmR-gp11** plasmid is an engineered bacterial artificial chromosome, in which all codons of *gp11* have undergone synonymous mutations.


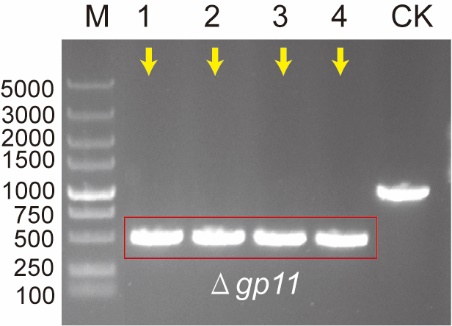


**Fig. S3.** PCR analysis of *gp11* deletion. The four candidate *gp11*-deficient T7 phages were checked by colony PCR. The correct phages are indicated by yellow arrows. Wild-type T7 phage was used as the control (CK).


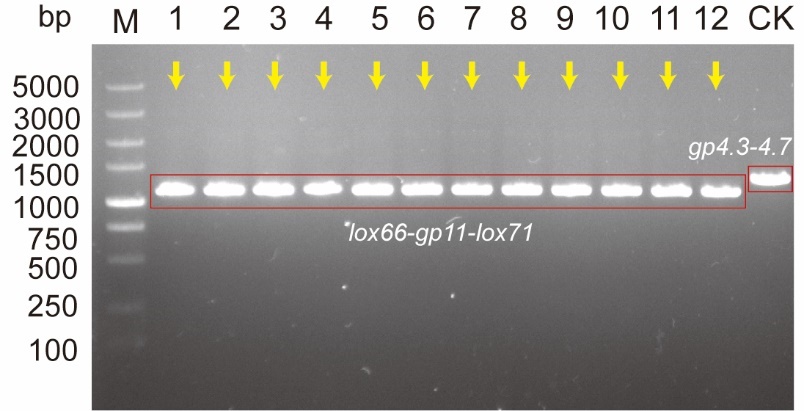


**Fig. S4.** Verification of recombination accuracy with *gp11* as the selection marker by PCR. Twelve T7 recombinant phages were randomly selected for evaluation of recombination accuracy via colony PCR. The correct phages are indicated by yellow arrows. Wild-type T7 phage was used as the control (CK).


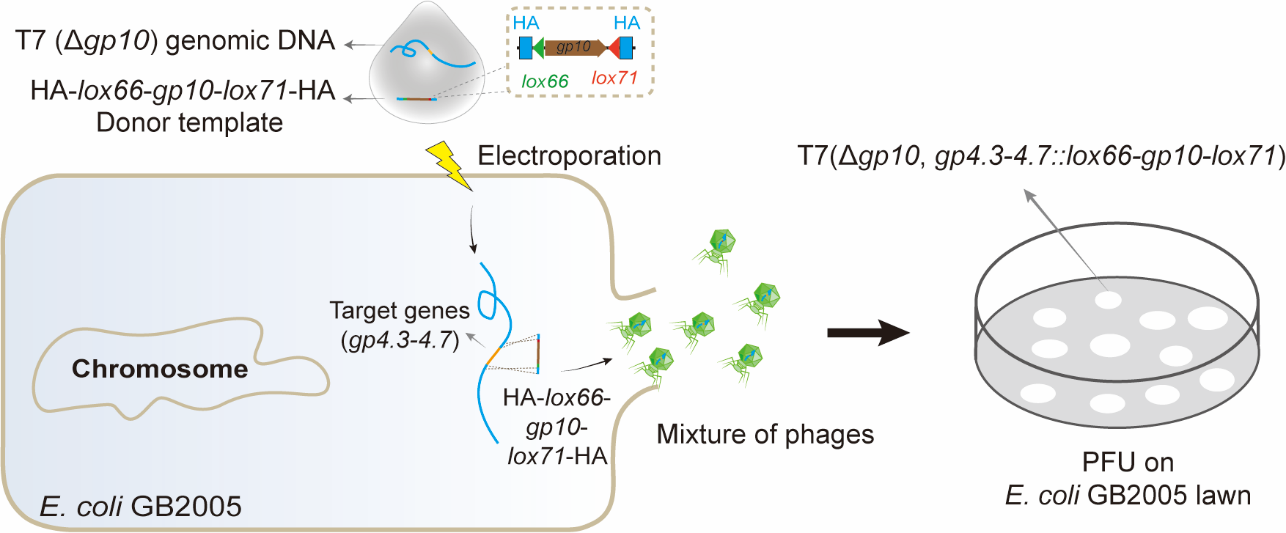


**Fig. S5.** Schematic diagram of eDCHR. The HA-*lox66-gp10-lox71*-HA donor template is co-electroporated with T7 (Δ*gp10*) genomic DNA into *E. coli* GB2005. The targeted genes (*gp4.3*–*4.7*) are replaced by the donor template via homologous recombination. The recombinant T7 (Δ*gp10*, *gp4.3*–*4.7::lox66-gp10-lox71*) phages are isolated on *E. coli* GB2005 lawns. White dots indicate plaques.


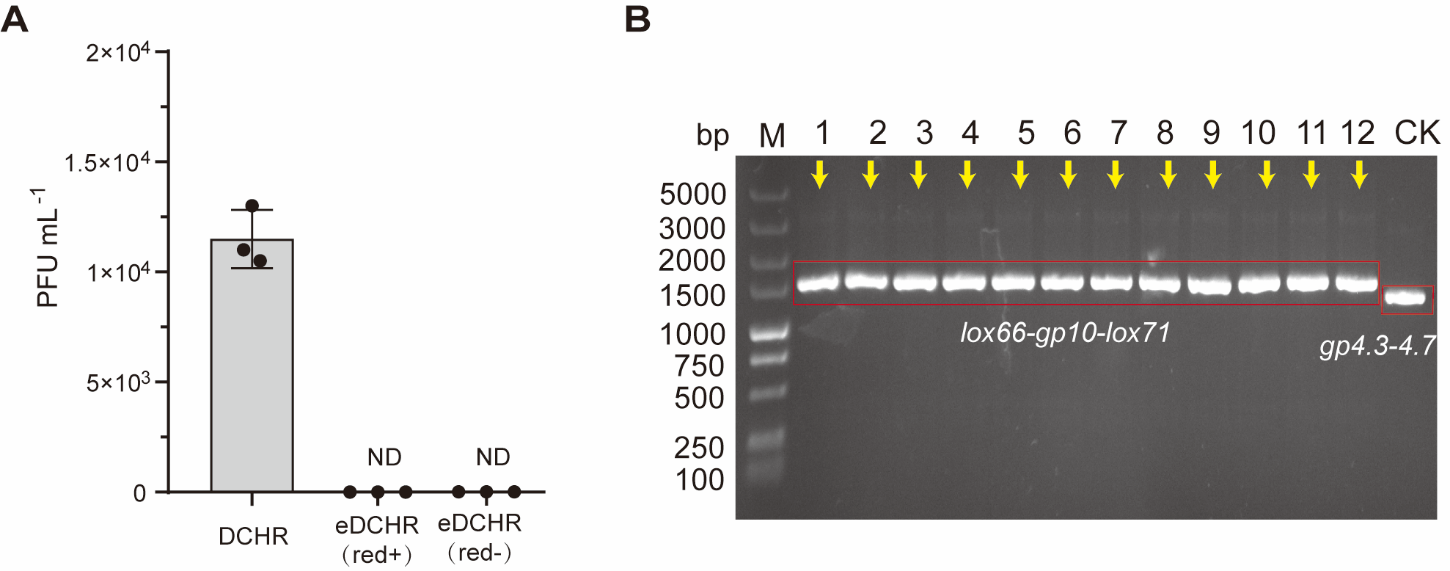


**Fig. S6.** Recombination efficiency of DCHR and eDCHR in *E. coli* MG1655. (A) Comparison of recombination efficiency between DCHR and eDCHR with 500-bp homology arms in *E. coli* MG1655. The pSC101-BAD-*gbaA-tetR* plasmid carrying the λ-Red recombination system was transferred into *E. coli* MG1655 for eDCHR recombineering [[1](#_ENREF_1)]. Recombination efficiencies of eDCHR without (Red-) and with (Red+) the λ-Red recombination system were determined. Experiments were performed in triplicate (*n* = 3). Data are presented as mean ± S.D. with error bars shown. ND, no recombinant phages were detected. (B) PCR verification of recombinant phages. Twelve randomly selected T7 recombinant phages were analyzed by colony PCR. Correct recombinant phages are indicated by yellow arrows. Wild-type T7 phage was used as the control (CK).


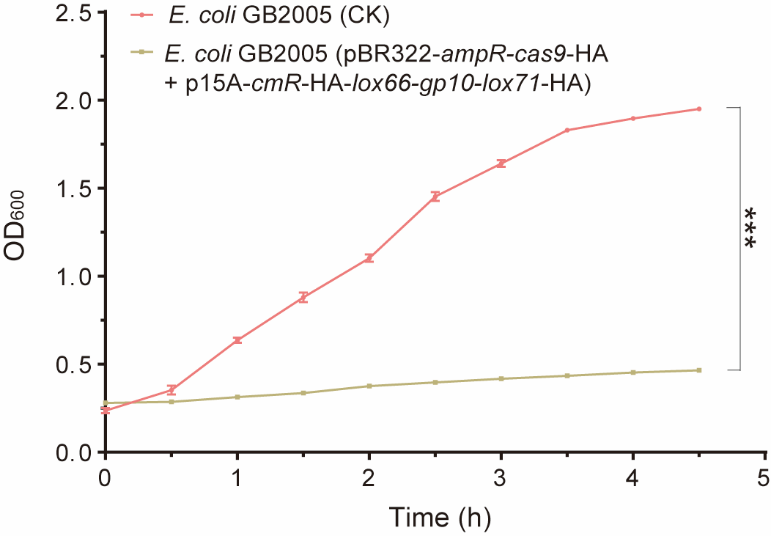


**Fig. S7.** Growth curve of *E. coli* GB2005 harboring the pBR322-*ampR-cas9*-HA and p15A-*cmR*-HA-*lox66-gp10-lox71*-HA plasmids*.* The *E. coli* GB2005 harboring the pBR322-*ampR-cas9*-HA with empty p15A plasmids was used as the control (CK)*.* OD_600_ values were measured every 0.5 hours until 4.5 hours. Experiments were performed in triplicate (*n* = 3). Data are presented as mean ± S.D. with error bars shown. Statistical analysis comparing two groups was performed using a two-sided Student’s *t*-test. **P*<0.05; ***P*<0.01; ****P*<0.001; ns, not significant.


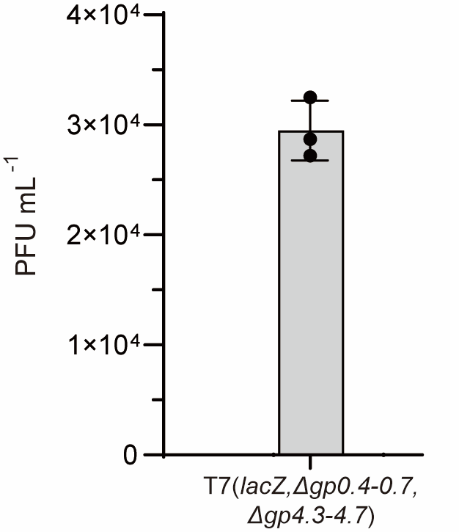


**Fig. S8.** Recombination efficiency of DCHR in inserting the *lacZ* gene.


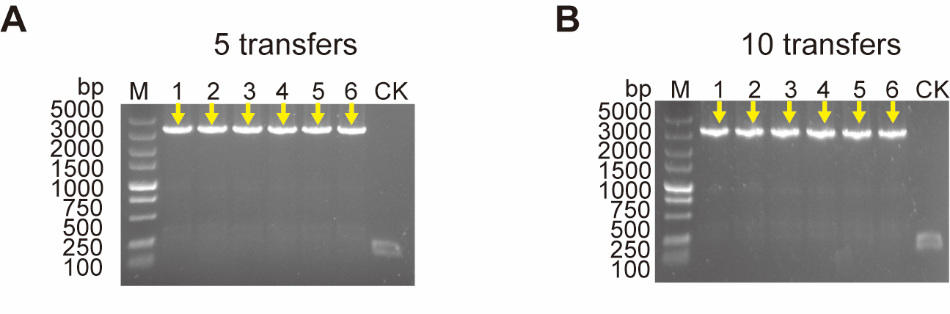


**Fig. S9.** PCR amplification of the *lacZ* gene in the genomes of recombinant T7 (*lacZ*) progeny phages. (A) PCR verification of the *lacZ* in six progeny phages after 5 transfers. (B) PCR verification of the *lacZ* in six progeny phages after 10 transfers. Wild-type T7 phage was used as the control (CK). Correct progeny phages are indicated by yellow arrows.


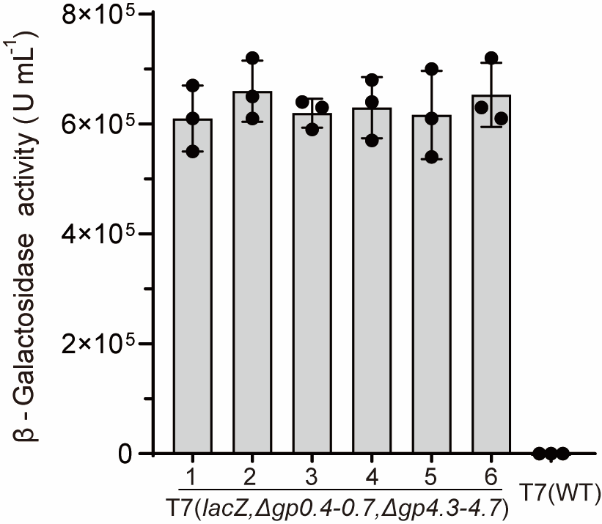


**Fig. S10.** Determination of β-galactosidase activity in six recombinant T7 (*lacZ*) progeny phages after 10 transfers. T7 (WT) represents the wild-type T7 phage.

**Table S1.** Bacteria and phages used in this work.

| **Strains** | **Genotype or relevant features** |
| --- | --- |
| *E. coli* GB2005 [[2](#_ENREF_2)] | (HS996, derived from DH10B, Δ*recET*, Δ*ybcC*), The endougenous *recET* locus and DLP12 prophage *ybcC*, which encodes a putative exonuclease, were deleted |
| *E. coli* GB05-red [[2](#_ENREF_2)] | (GB2005, araC-BAD-αβγA) lambda *red* operon and *recA* under P_BAD_ promoter were inserted at the *ybcC* locus |
| *E. coli* GB05-dir [[3](#_ENREF_3)] | (GB2005, araC-BAD-ETgA) *recE, recT* and *recA* under P_BAD_ promoter were inserted at the *ybcC* locus |
| *E. coli* Nissle 1917 | Accession No. CP007799 |
| *E. coli* MG1655 | Accession No. NC_000913 |
| T7 phage | Accession No. NC_001604 |

**Table S2.** Nonessential gene list in T7 phage genome [[4](#_ENREF_4), [5](#_ENREF_5)].

| **Nonessential gene regions** | **Start** | **End** | **Genes** | **sizes (bp)** |
| --- | --- | --- | --- | --- |
| *gp0.4-0.7* | 1,257 | 2,737 | *gp0.4, gp0.5, gp0.6A, gp0.6B, gp0.7A* | 1,481 |
| *gp1.4* | 7,609 | 7,764 | *gp1.4* | 156 |
| *gp1.7* | 8,198 | 8,733 | *gp1.7* | 536 |
| *gp3.8* | 11,268 | 11,558 | *gp3.8* | 291 |
| *gp4.3-4.7* | 13,279 | 14,297 | *gp4.3, gp4.5, gp4.7* | 1,019 |
| *gp5.3* | 16,484 | 16,821 | *gp5.3* | 338 |
| *gp7.7* | 20,109 | 20,208 | *gp7.7* | 100 |
| *gp19.5* | 39,430 | 39,628 | *gp19.5* | 199 |

**Table S3.** Oligonucleotides for construction of cassettes.

| **Name** | **sequence (5’-3’)** | **Template** | **Purpose** |
| --- | --- | --- | --- |
| lox66-gp10-F | aacgacactgacttctgacaggattcttgatAAGCTTGGATCCTACCGTTCGTATAATGTATGCTATAC | Genomic DNA of T7 phage | Amplification of *gp10 and* construction of the *lox66-gp10-lox71* cassette |
| lox71-gp10-R | tctcctattgattatcgtgacttaacaatctcttcatatgaaacaactTACCGTTCGTATAGCATACA |  |  |
| 500HA-D1-F1 | GCTCGCTAAGCTGGCCTACA | Genomic DNA of T7 phage | Construction of the HA-*lox66-gp10-lox71*-HA donor template with 500-bp HA targeting *gp4.3-4.7* |
| 500HA-D1-R1 | ATCAAGAATCCTGTCAGAAG |  |  |
| 500HA-D1-F2 | AGTTGTTTCATATGAAGAGATTG |  |  |
| 500HA-D1-R2 | TATTCTTCACCCTGCTCTTC |  |  |
| 200HA-D1-F | TCAGCAAGGCGATATGCCTA | The *lox66-gp10-lox71* cassette | Construction of the HA-*lox66-gp10-lox71*-HA donor template with 200-bp HA targeting *gp4.3-4.7* |
| 200HA-D1-R | CGGCTTCCAGCGCATCCAGA |  |  |
| 50HA-D1-F | AGAGTCAACAGACTGGTCCA | The *lox66-gp10-lox71* cassette | Construction of the HA-*lox66-gp10-lox71*-HA donor template with 50-bp HA targeting *gp4.3-4.7* |
| 50HA-D1-R | TTTCTCCTATTGATTATCGT |  |  |

Underlined sequences are homology arms for recombineering.

**Table S4.** Oligonucleotides for construction of plasmids.

| **Name** | **sequence (5’-3’)** | **Template** | **Purpose** |
| --- | --- | --- | --- |
| BAC-gp10-F | TTATTCGACCTTAAATACGA | The artificially synthesized *gp10** fragment | Construction of the pBAC-*cmR-gp10** plasmid |
| BAC-gp10-R | ATGGCATCCATGACCGGAGG |  |  |
| BAC-F | CATCTGTTGACCTCCGGTCATGGATGCCATATGTATATCTCCTTCTTAAA | pBAC-*cmR*-BR322-*ccdB-hyg*[[6](#_ENREF_6)] |  |
| BAC-R | GCAGGCGCAGTCGTATTTAAGGTCGAATAATGTTCGGCCTTGAATTGATCAT |  |  |
| pBR322-cas9-R | TTCGTCCATTTGACAGGCAC | pBR322-*amp-ccdB-rpsl* [[6](#_ENREF_6)] | Construction of the pBR322-*ampR-cas9-gp10* plasmid |
| pBR322-cas9-F | CTGCAGTTGCTGGCGTTTTT |  |  |
| Cas9-F | GTGCCTGTCAAATGGACGAAGCAGGGATTCTGCAAACCCT | The artificially synthesized *cas9* fragment |  |
| Cas9-R | ATATACGAGTCAATGAAAGAAAAAAGCACCGACTCGGTGCCACTTTTTCAAGTTGATAA |  |  |
| 500HA-gp10-F1 | CTTTCATTGACTCGTATATC | Genomic DNA of T7 phage |  |
| 500HA-gp10-R1 | TTGAGGCCACCCCCAGCAGATATCGAATTCATGTATATCTCCTTCTTAAA |  |  |
| 500HA-gp10-F2 | GAATTCGATATCTGCTGGGG |  |  |
| 500HA-gp10-R2 | AAAAACGCCAGCAACTGCAGACATTAGGGATAAATAGTCG |  |  |
| 500HA-gp11-F1 | TGGTACCGCTCGTGAGGGCA | Genomic DNA of T7 phage | Construction of the pBR322-*ampR-cas9-gp11* plasmid |
| 500HA-gp11-R1 | GTGCCATTAGAGCCTCCTTATTTATTAATGATAGTTCCTCCTTTCAGCAA |  |  |
| 500HA-gp11-F2 | CATTAATAAATAAGGAGGCT |  |  |
| 500HA-gp11-R2 | AAAAACGCCAGCAACTGCAGACCACCACGAACGTTAATCA |  |  |
| BAC-kan-F | GATGCTCGATGAGTTTTTCTAAACGTCTCATTTTCGCCAAAAG | pBAC-*cm*-BR322-*ccdB-hyg*[[6](#_ENREF_6)] | Construction of the pBAC-*kanR-cas9*-HA plasmid |
| BAC-kan-R | TTCGTCCATTTGACAGGCACATTATGCATCCGCGGTGAGATCCGGCTGCTAACAA |  |  |
| Cas9-F1 | GTGCCTGTCAAATGGACGAAGCAGGGATTCTGCAAACCCT | The artificially synthesized *cas9* fragment |  |
| Cas9-R1 | CTGCAGAAAAAAGCACCGACTCGGTGCCACTTTTTCAAGTTGATAA |  |  |
| Kan-F | CACCGAGTCGGTGCTTTTTTCTGCAGATCCTCTACGCCGGACGCAT | Kanamycin resistance gene |  |
| Kan-R | TTAGAAAAACTCATCGAGCATC |  |  |
| p15A-F | GAATACCTCTTCGCGGAATAAGGTCCAGTATCGCAGACGGCACAACCTCGCAGAAAGGCCCACCCGA | p15A-*cm-ccdB* [[2](#_ENREF_2)] | Construction of the p15A-*cmR*-HA-*lox66-gp10-lox71*-HA plasmid containing the recognition sites of Cas9 nuclease and 50-bp HA targeting the *gp4.3-4.7* region. |
| p15A-R | CGAGCCCTCTTCGCGGAATAAGGTCCAGGTAACGCTTTCCAACCTTGACGAAGAAAGGCCCACCCGT |  |  |
| p50HA-D1-F | AGAGTCAACAGACTGGTCCAACGACACTGACTTCTGACAGGATTCTTGAT | The HA-*lox66-gp10-lox71*-HA donor template |  |
| p50HA-D1-R | TTTCTCCTATTGATTATCGTGACTTAACAATCTCTTCATATGAAACAACT |  |  |
| p200HA-D1-F1 | GTTACCTGGACCTTATTCCGCGAAGAGGTCAGCAAGGCGATATGCCT | The HA-*lox66-gp10-lox71*-HA donor template | Construction of the p15A-*cmR*-HA-*lox66-gp10-lox71*-HA plasmid containing the recognition sites of Cas9 nuclease and 200-bp HA targeting the *gp4.3-4.7* region. |
| p200HA-D1-R1 | CGATACTGGACCTTATTCCGCGAAGAGGCGGCTTCCAGCGCATCCAGA |  |  |
| p500HA-D1-F | GTTACCTGGACCTTATTCCGCGAAGAGGGCTCGCTAAGCTGGCCTAC | The HA-*lox66-gp10-lox71*-HA donor template | Construction of the p15A-*cmR*-HA-*lox66-gp10-lox71*-HA plasmid containing the recognition sites of Cas9 nuclease and 500-bp HA targeting the *gp4.3-4.7* region. |
| p500HA-D1-R | CGATACTGGACCTTATTCCGCGAAGAGGTATTCTTCACCCTGCTCTTC |  |  |
| p500HA-D2-F | CTGGACCTTATTCCGCGAAGAGGTCTCACAGTGTACGGACCTA | The HA-*lox66-gp10-lox71*-HA donor template | Construction of the p15A-*cmR*-HA-*lox66-gp10-lox71*-HA plasmid containing the recognition sites of Cas9 nuclease and 50-bp HA targeting the *gp0.4-0.7* region. |
| p500HA-D2-R | CGATACTGGACCTTATTCCGCGAAGAGGCAGCCAGAGTGTTGAACGGG |  |  |
| gp9-point mutation-F1 | GTTACCTGGACCTTATTCCGCGAAGAGGCTGCCTCTGAGCAACTTGGT | Genomic DNA of T7 phage | Construction of the HA p15A-*cmR*-HA*-gp10*-HA plasmid containing the recognition sites of Cas9 nuclease and 500-bp HA targeting *gp9.* |
| gp9-point mutation-R1 | TAGAAGTTCGAATCGATTAC |  |  |
| gp9-point mutation-F2 | GTAATCGATTCGAACTTCTAATAGACTTCGAAATTAATAC |  |  |
| gp9-point mutation-R2 | CGATACTGGACCTTATTCCGCGAAGAGGGGCTGATACCACCCTTCAAG |  |  |
| lacZ-F | TGCTGGTGCAGTGGTTTTCAAAGTGGAGTAAGGATCCTTGACAATTAATCATCGGCTCGTATAATGTGTGGAATTTCACACAGGAGGAATTCATATGACTATGATTACGGATTCTCTG | Genomic DNA of *E. coli* Nissle 1917 | Construction of the p15A-cmR-HA-*gp10*-*lacZ*-HA plasmid containing the recognition sites of Cas9 nuclease and 500-bp HA targeting *gp10.* |
| lacZ-R | TAGCAGCGACCGTTGAGGCCACCCCCAGCATTATTTTTGACACCAGACCA |  |  |
| gp10-lacZ-F1 | GTTACCTGGACCTTATTCCGCGAAGAGGCTGCCTCTGAGCAACTTGGT | Genomic DNA of T7 phage |  |
| gp10-lacZ-R1 | TTACTCCACTTTGAAAACCA |  |  |
| gp10-lacZ-F2 | TGCTGGGGGTGGCCTCAACG |  |  |
| gp10-lacZ-R2 | CGATACTGGACCTTATTCCGCGAAGAGGGGCTGATACCACCCTTCAAG |  |  |

Underlined sequences are homology arms for recombineering.

**Table S5.** List of gene sequences.

| **genes** | **sequence (5’-3’)** |
| --- | --- |
| *gp10**  (1038 bp) | ATGGCATCCATGACCGGAGGTCAACAGATGGGCACAAATCAGGGAAAGGGCGTGGTAGCAGCAGGTGACAAGCTGGCTTTGTTTCTCAAAGTCTTCGGTGGCGAGGTGCTCACAGCCTTTGCACGCACGTCTGTCACGACATCCCGTCATATGGTGCGCTCTATTTCTTCCGGCAAGAGCGCACAATTTCCTGTACTCGGCCGTACACAAGCTGCCTACCTCGCACCGGGTGAAAATCTGGATGACAAGCGCAAAGATATTAAGCATACGGAAAAAGTTATTACGATCGATGGCCTGCTCACCGCAGATGTACTCATCTACGACATCGAAGATGCCATGAATCATTATGATGTACGTTCAGAATACACGTCCCAACTCGGCGAGTCCCTCGCCATGGCAGCCGACGGCGCCGTACTCGCAGAAATCGCGGGCCTCTGCAATGTCGAGTCTAAGTACAACGAAAATATTGAAGGTCTGGGCACAGCAACGGTTATCGAAACGACACAAAATAAAGCGGCTCTAACGGATCAGGTAGCCCTCGGCAAAGAAATCATCGCCGCACTCACAAAAGCACGCGCCGCACTCACGAAAAATTACGTACCGGCAGCAGATCGCGTCTTTTATTGCGATCCAGACTCCTATTCAGCCATCCTCGCTGCTCTCATGCCGAATGCTGCTAATTATGCAGCACTCATCGATCCTGAAAAAGGCTCCATTCGTAATGTAATGGGCTTCGAAGTAGTTGAGGTACCTCATCTGACGGCAGGCGGCGCAGGCACGGCACGCGAAGGTACAACAGGCCAGAAACACGTGTTTCCGGCGAACAAGGGCGAAGGCAACGTGAAAGTAGCAAAAGATAATGTAATTGGTCTGTTTATGCACCGTTCCGCCGTTGGCACAGTAAAACTCCGCGATCTTGCACTCGAACGTGCACGTCGCGCAAATTTTCAAGCCGATCAGATCATTGCAAAATATGCTATGGGTCACGGCGGCTTGCGTCCTGAGGCAGCAGGCGCAGTCGTATTTAAGGTCGAATAA |
| *gp11**  (591 bp) | ATGCGTTCTTATGACATGAATGTAGAAACCGCAGCAGAATTGTCTGCCGTAAATGATATCCTCGCTTCAATTGGAGAGCCACCTGTGTCTACCCTCGAGGGAGATGCAAATGCTGACGCTGCCAATGCACGTCGAATCCTGAATAAAATCAATCGTCAAATCCAGTCACGAGGTTGGACCTTTAATATCGAAGAGGGGATTACCCTCCTTCCAGACGTATATTCTAATCTCATCGTGTATTCAGATGATTACTTGTCTCTGATGTCCACCTCTGGACAGTCTATTTATGTGAATCGTGGAGGTTACGTATACGATCGTACTTCTCAGTCTGATCGGTTCGATTCCGGAATCACCGTAAATATCATCCGACTGCGTGATTATGACGAAATGCCAGAATGTTTTCGCTATTGGATCGTGACTAAAGCCTCTCGACAGTTTAATAATCGTTTTTTCGGTGCTCCTGAGGTGGAAGGAGTGCTGCAGGAGGAAGAGGACGAAGCACGTCGCCTGTGTATGGAATACGAAATGGATTATGGAGGCTATAACATGCTCGACGGTGACGCTTTTACCTCAGGACTGCTCACCCGTTAA |
| *lacZ* from *E. coli* Nissle 1917  (3075 bp, Protein ID: AID77448.1) | ATGACTATGATTACGGATTCTCTGGCCGTCGTATTACAACGTCGTGACTGGGAAAACCCTGGCGTTACCCAACTTAATCGCCTTGCGGCACATCCCCCTTTCGCCAGCTGGCGTAATAGCGAAGAGGCCCGCACCGATCGCCCTTCCCAACAGTTGCGCAGCCTGAATGGCGAATGGCGCTTTGCCTGGTTTCCGGCACCAGAAGCGGTGCCGGAAAGCTGGCTGGAGTGCGATCTTCCTGACGCCGATACTGTCGTCGTCCCCTCAAACTGGCAGATGCACGGTTACGATGCGCCTATCTACACCAACGTGACCTATCCCATTACGGTCAATCCGCCGTTTGTTCCCGCGGAGAATCCGACAGGTTGTTACTCGCTCACATTTAATATTGATGAAAGCTGGCTACAGGAAGGCCAGACGCGAATTATTTTTGATGGCGTTAACTCGGCGTTTCATCTGTGGTGCAACGGGCGCTGGGTCGGTTACGGCCAGGACAGCCGTTTGCCGTCTGAATTTGACCTGAGCGCATTTTTACGCGCCGGAGAAAACCGCCTCGCGGTGATGGTGCTGCGCTGGAGTGACGGCAGTTATCTGGAAGATCAGGATATGTGGCGGATGAGCGGCATTTTCCGTGACGTCTCGTTGCTGCATAAACCGACCACGCAAATCAGCGATTTCCAAGTTACCACTCTCTTTAATGATGATTTCAGCCGCGCGGTACTGGAGGCAGAAGTTCAGATGTACGGCGAGCTGCGCGATGAACTGCGGGTGACGGTTTCTTTGTGGCAGGGTGAAACGCAGGTCGCCAGCGGCACCGCGCCTTTCGGCGGTGAAATTATCGATGAGCGTGGCGGTTATGCCGATCGCGTCACACTACGCCTGAACGTTGAAAATCCGGAACTGTGGAGCGCCGAAATCCCGAATCTCTATCGTGCAGTGGTTGAACTGCACACCGCCGACGGCACGCTGATTGAAGCAGAAGCCTGCGACGTCGGTTTCCGCGAGGTGCGGATTGAAAATGGTCTGCTGCTGCTGAACGGCAAGCCGTTGCTGATTCGCGGCGTTAACCGTCACGAGCATCATCCTCTGCATGGTCAGGTCATGGATGAGCAGACGATGGTGCAGGATATCCTGCTGATGAAGCAGAACAACTTTAACGCCGTGCGCTGTTCGCATTATCCGAACCATCCGCTGTGGTACACGCTGTGCGACCGCTACGGCCTGTATGTGGTGGATGAAGCCAATATTGAAACCCACGGCATGGTGCCAATGAATCGTCTGACCGATGATCCGCGCTGGCTACCCGCGATGAGCGAACGCGTAACGCGGATGGTGCAGCGCGATCGTAATCACCCGAGTGTGATCATCTGGTCGCTGGGGAATGAATCAGGCCACGGCGCTAATCACGACGCGCTGTATCGCTGGATCAAATCTGTCGATCCTTCCCGCCCGGTACAGTATGAAGGCGGCGGAGCCGACACCACGGCCACCGATATTATTTGCCCGATGTACGCGCGCGTGGATGAAGACCAGCCCTTCCCGGCGGTGCCGAAATGGTCCATCAAAAAATGGCTTTCGCTGCCTGGAGAAATGCGCCCGCTGATCCTTTGCGAATATGCCCACGCGATGGGTAACAGTCTTGGCGGCTTCGCTAAATACTGGCAGGCGTTTCGTCAGTACCCCCGTTTACAGGGCGGCTTCGTCTGGGACTGGGTGGATCAGTCGCTGATTAAATATGATGAAAACGGCAACCCGTGGTCGGCTTACGGCGGTGATTTTGGCGATACGCCGAACGATCGCCAGTTCTGTATGAACGGTCTGGTCTTTGCCGACCGCACGCCGCATCCGGCGCTGACGGAAGCAAAACACCAACAGCAGTATTTCCAGTTCCGTTTATCCGGGCGAACCATCGAAGTGACCAGCGAATACCTGTTCCGTCATAGCGATAACGAGTTCCTGCACTGGATGGTGGCACTGGATGGCAAGCCGCTGGCAAGCGGTGAAGTGCCTCTGGATGTTGGCCCGCAAGGTAAGCAGTTGATTGAACTGCCTGAACTGCCGCAGCCGGAGAGCGCCGGACAACTCTGGCTAACGGTACGCGTAGTGCAACCAAACGCGACCGCATGGTCAGAAGCCGGACACATCAGCGCCTGGCAGCAATGGCGTCTGGCGGAAAACCTCAGCGTGACACTCCCCTCCGCGTCCCACGCCATCCCTCAACTGACCACCAGCGGAACGGATTTTTGCATCGAGCTGGGTAATAAGCGTTGGCAATTTAACCGCCAGTCAGGCTTTCTTTCACAGATGTGGATTGGCGATGAAAAACAACTGCTGACCCCGCTGCGCGATCAGTTCACCCGTGCGCCGCTGGATAACGACATTGGCGTAAGTGAAGCGACCCGCATTGACCCTAACGCCTGGGTCGAACGCTGGAAGGCGGCGGGCCATTACCAGGCCGAAGCGGCGTTGTTGCAGTGCACGGCAGATACACTTGCCGACGCGGTGCTGATTACAACCGCCCACGCGTGGCAGCATCAGGGGAAAACCTTATTTATCAGCCGGAAAACCTACCGGATTGATGGGCACGGTGAGATGGTCATCAATGTGGATGTTGCGGTGGCAAGCGATACACCGCATCCGGCGCGGATTGGCCTGACCTGCCAGCTGGCGCAGGTCTCAGAGCGGGTAAACTGGCTCGGCCTGGGGCCGCAAGAAAACTATCCCGACCGCCTTACTGCAGCCTGTTTTGACCGCTGGGATCTGCCATTGTCAGACATGTATACCCCGTACGTCTTCCCGAGCGAAAACGGTCTGCGCTGCGGGACGCGCGAATTGAATTATGGCCCACACCAGTGGCGCGGCGACTTCCAGTTCAACATCAGCCGCTACAGCCAACAACAACTGATGGAAACCAGCCATCGCCATCTGCTGCACGCGGAAGAAGGCACATGGCTGAATATCGACGGTTTCCATATGGGGATTGGTGGCGACGACTCCTGGAGCCCGTCAGTATCGGCGGAATTCCAGCTGAGCGCCGGTCGCTACCATTACCAGTTGGTCTGGTGTCAAAAATAA |

**Supplementary references**

[1] Wang J, Sarov M, Rientjes J, Fu J, Hollak H, Kranz H, et al. An improved recombineering approach by adding RecA to lambda Red recombination. Mol Biotechnol 2006;32:43-53. <https://dx.doi.org/10.1385/mb:32:1:043>.

[2] Wang H, Bian X, Xia L, Ding X, Müller R, Zhang Y, et al. Improved seamless mutagenesis by recombineering using *ccdB* for counterselection. Nucleic Acids Res 2014;42:e37. <https://dx.doi.org/10.1093/nar/gkt1339>.

[3] Fu J, Bian X, Hu S, Wang H, Huang F, Seibert PM, et al. Full-length RecE enhances linear-linear homologous recombination and facilitates direct cloning for bioprospecting. Nat Biotechnol 2012;30:440-446. <https://dx.doi.org/10.1038/nbt.2183>.

[4] Yuan S, Shi J, Jiang J, Ma Y. Genome-scale top-down strategy to generate viable genome-reduced phages. Nucleic Acids Res 2022;50:13183-13197. <https://dx.doi.org/10.1093/nar/gkac1168>.

[5] Zhang H, Zhu R, Wang Z, He R, Zhang Y, Luan J, et al. Programming virulent bacteriophages by developing a multiplex genome engineering method. mBio 2025;16:e0358224. <https://dx.doi.org/10.1128/mbio.03582-24>.

[6] Wang H, Li Z, Jia R, Hou Y, Yin J, Bian X, et al. RecET direct cloning and Redαβ recombineering of biosynthetic gene clusters, large operons or single genes for heterologous expression. Nat Protoc 2016;11:1175-1190. <https://dx.doi.org/10.1038/nprot.2016.054>.
